# Insights into the Histone Acetylation-Mediated Regulation of the Transcription Factor Genes That Control the Embryogenic Transition in the Somatic Cells of Arabidopsis

**DOI:** 10.3390/cells11050863

**Published:** 2022-03-02

**Authors:** Joanna Morończyk, Agnieszka Brąszewska, Barbara Wójcikowska, Karolina Chwiałkowska, Katarzyna Nowak, Anna M. Wójcik, Mirosław Kwaśniewski, Małgorzata D. Gaj

**Affiliations:** 1 Institute of Biology, Biotechnology and Environmental Protection, Faculty of Natural Sciences, University of Silesia in Katowice, 40-007 Katowice, Poland; joanna.moronczyk@us.edu.pl (J.M.); agnieszka.braszewska@us.edu.pl (A.B.); barbara.wojcikowska@us.edu.pl (B.W.); katarzyna.nowak@us.edu.pl (K.N.); anna.wojcik@us.edu.pl (A.M.W.); 2 Centre for Bioinformatics and Data Analysis, Medical University of Bialystok, 15-269 Bialystok, Poland; karolina.chwialkowska@umb.edu.pl (K.C.); miroslaw.kwasniewski@umb.edu.pl (M.K.)

**Keywords:** Arabidopsis, auxin, histone acetylation, somatic embryogenesis, transcription factors, trichostatin A

## Abstract

Somatic embryogenesis (SE), which is a process that involves the in vitro-induced embryogenic reprogramming of plant somatic cells, requires dynamic changes in the cell transcriptome. These changes are fine-tuned by many genetic and epigenetic factors, including posttranslational histone modifications such as histone acetylation. Antagonistically acting enzymes, histone acetyltransferases (HATs) and deacetylases (HDACs), which control histone acetylation in many developmental processes, are believed to control SE. However, the function of specific HAT/HDACs and the genes that are subjected to histone acetylation-mediated regulation during SE have yet to be revealed. Here, we present the global and gene-specific changes in histone acetylation in Arabidopsis explants that are undergoing SE. In the TSA (trichostatin A)-induced SE, we demonstrate that H3 and H4 acetylation might control the expression of the critical transcription factor (*TF*) genes of a vital role in SE, including *LEC1*, *LEC2* (*LEAFY COTYLEDON 1; 2*), *FUS3* (*FUSCA 3*) and *MYB118* (*MYB DOMAIN PROTEIN 118*). Within the HATs and HDACs, which mainly positively regulate SE, we identified HDA19 as negatively affecting SE by regulating *LEC1*, *LEC2* and *BBM*. Finally, we provide some evidence on the role of HDA19 in the histone acetylation-mediated regulation of *LEC2* during SE. Our results reveal an essential function of histone acetylation in the epigenetic mechanisms that control the *TF* genes that play critical roles in the embryogenic reprogramming of plant somatic cells. The results implicate the complexity of Hac-related gene regulation in embryogenic induction and point to differences in the regulatory mechanisms that are involved in auxin- and TSA-induced SE.

## 1. Introduction

Chromatin, which is a complex of DNA and histones, plays a central role in controlling the gene expression at the transcriptional level by modulating the accessibility of DNA to the transcription factors (TFs) [1]. Dynamic changes in the chromatin structure are orchestrated by epigenetic modifications, including DNA methylation and the posttranslational modifications of histones such as acetylation, methylation and phosphorylation [2].

In contrast to DNA methylation, the role of the histone modification, including histone acetylation (Hac), in regulating gene expression is less recognised, primarily because of the limited number of available analytical tools [3]. In recent years, new molecular methods applied in epigenetics have contributed to significant progress in revealing experimental evidence about the essential role of histone modifications, including Hac, in regulating the gene expression in various organisms, including plants [4,5]. During the Hac-mediated regulation of gene expression, histone acetyltransferases (HATs) neutralise the positive charge of lysine in the histone tails, and that, in turn, promotes an “open” and transcriptionally permissive chromatin structure [6]. Conversely, histone deacetylases (HDACs) restore the positive charge of the lysine in histones, which triggers chromatin condensation and negatively affects gene expression [7]. In order to activate or repress the target gene expression, HATs and HDACs act as a part of the transcriptional complexes and control many developmental processes in plants [8]. In addition to functioning in the transcriptional complexes, HATs and HDACs might also control the de/acetylation state of the non-histone proteins [8,9]. 

In eukaryotes, epigenetic modifications control diverse developmental processes, including the extensive transcriptome reprogramming that is associated with changes in the cell fate [10,11]. It is believed that dedifferentiated cells have an open state of chromatin that enables re-differentiation, while compact chromatin is required to stabilise the gene expression and to maintain the identity of the differentiated cells [12,13]. 

During somatic embryogenesis (SE), a plant-specific developmental process, the embryogenic transition that is induced in the in vitro-cultured and differentiated plant somatic cells results in embryo-like structures, i.e., somatic embryos that efficiently regenerate into plants. SE-based plant regeneration systems are widely used in biotechnology for the micropropagation and genetic transformation of different plant species [14]. Thus, understanding the molecular processes that control the embryogenic switch in somatic plant cells is of interest to current plant biotechnology. The extensive reprogramming of the cell transcriptome that is associated with SE induction involves erasing or bypassing the existing cell fate memory under the control of epigenetic modifications [15,16]. Most reports on the epigenetic control of SE concern DNA methylation [17]. Both the hypo- and hypermethylation of DNA have been attributed to SE induction, and changes in DNA methylation have also been postulated to regulate the *TF* genes that have a function in the embryogenic response [18,19,20]. To regulate gene expression, DNA methylation interplays with histone modifications, and, within them, histone methylation has been the most frequently investigated in in vitro-cultured plant tissue [21,22,23]. Histone methylation, which is catalysed by the POLYCOMB REPRESSIVE COMPLEX 2 (PRC2), has been reported to control several of the *TF* genes that are involved in cell differentiation and SE induction [24]. 

The function of Hac in regulating genes during in vitro-induced plant morphogenesis remains much less recognised and mainly only indirect evidence has been reported. The impaired function of histone acetyltransferases and deacetylases negatively affect the morphogenic responses, including callus formation and shoot regeneration in in vitro-cultured plant cells/tissue [25,26,27,28]. Moreover, the differential expression of the *HAT* and *HDAC* genes in embryogenic cultures of Arabidopsis and *Hevea brasiliensis* indirectly implies that dynamic epigenetic changes accompany SE induction [29,30]. Similarly, microspore-derived embryos of *Brassica napus* had an elevated level of *BnHAT*, which corresponded to an increased accumulation of the H3ac and H4ac epigenetic marks [31]. In *Pinus radiata*, the higher capacity for the in vitro shoot organogenesis of immature needles than for mature needles was attributed to a higher level of H4ac [32].

Experiments with trichostatin A (TSA), which is an inhibitor of the zinc-dependent HDACs [33], provided evidence on the role of Hac in the regulation of SE in plants. Accordingly, TSA treatment increased the embryogenic responses that were induced in vitro in cultures of various plants, including conifers, cereals and dicots [34,35,36]. The SE-promoting effect of TSA treatment has also been indicated in the model plant Arabidopsis and its TSA-induced seedlings and explants developed somatic embryos on auxin-free media [37,38]. It is worth noting that the TSA-induced embryogenic response in Arabidopsis was accompanied by changes in the gene expression, which suggests that Hac might contribute to SE induction via the deregulation of the genes that are involved in the embryogenic transition [37,38].

The genes that have an essential function in embryogenic induction encode several hormone- and stress-related transcription factors (TFs) that play central regulatory roles in both zygotic and somatic embryogenesis, including *LEAFY COTYLEDON 1;2 (LEC1* and *LEC2*), *FUSCA3* (*FUS3*), *BABY BOOM* (*BBM*), *AGAMOUS-LIKE15* (*AGL15*), *WUSCHEL* (*WUS*) and *MYB118* (*MYB DOMAIN PROTEIN 118*) [39,40]. *LEC1* encodes the HAP3 subunit of the CCAAT box-binding factor, while LEC2 represents a plant-specific family of TFs with a highly conserved B3 domain [41,42]. The *LEC1-* and *LEC2*-mediated mechanisms of SE induction involve activating the auxin biosynthesis *YUCCA* genes [43,44,45]. In SE induction, *LEC1* and *LEC2* are transcriptionally regulated by the BBM TF that is a member of the AINTEGUMENTA-LIKE (AIL) subfamily and contains two AP2/ERF (APETALA2/ethylene-responsive element) domains [46]. *LEC2* also interacts with *AGL15* of the MADS-box family of TFs to control the GA/ABA balance in SE induction [47,48]. *AGL15*, when ectopically expressed, leads to the formation of somatic embryos by controlling hormonal signalling during SE induction [49,50]. In zygotic embryogenesis (ZE), LEC2 targets *MYB118* of the *MYB* gene family in order to regulate the biosynthesis of the storage compounds and the *LATE EMBRYOGENESIS ABUNDANT (LEA*) genes [51,52]. In addition to *MYB118, MYB115,* by controlling the embryo storage products, also promotes an embryogenic transition in Arabidopsis [53]. Moreover, the *WUS* of the plant-specific homeobox superfamily of WOX TFs of a critical role in determining stem cell fate in the shoot apical meristem (SAM) of higher plants has also been attributed to the SE-regulatory network [54,55]. The early auxin gradient-dependent expression of *WUS* in the embryogenic callus of Arabidopsis was associated with somatic embryo development [56].

Evidence for the role of Hac in regulating the SE-associated *TF*s remains limited. It involves the role of HDA6 and HDA19 in the repression of the seed maturation *TF*s, including *LEC1*, *LEC2*, *FUS3* and *ABI3* (*ABA INSENSITIVE 3*) during seedling development [57,58,59]. Similarly, *LEC1*, *FUS3* and *ABI3* were deregulated in an *HDA6*/*19:RNAi* repression line of Arabidopsis that developed somatic embryos on the seedlings [37]. Further analysis is required to provide direct evidence on the role of Hac in controlling the genes of the SE-regulatory network that govern the embryogenic transition in plant somatic cells. 

Here, we gained insights into the global and gene-specific changes in the acetylation of the histones that are associated with the embryogenic transition that is induced in Arabidopsis explants in response to auxin and TSA treatment. The global changes in the acetylation of H3 histone in the SE-induced explants were monitored using ELISA and the immunohistochemical approaches. Moreover, in order to assess the role of Hac in controlling the SE-involved *TF*s, the H3/H4 acetylation status of the chromatin that was bound to *LEC1*, *LEC2*, *FUS3*, *MYB118*, *BBM*, *AGL15* and *WUS* was analysed using the ChIP-qPCR method. 

The results provide several experimental pieces of evidence on the function of Hac in controlling the embryogenic transition in plant somatic cells. The changes in the global Hac level, which are associated with the deregulation of numerous *HAT* and *HDAC* genes, were demonstrated in the SE-induced explants. The role of HDA19 in regulating the SE-*TF*s was shown and a correlation between the gene expression and H3/H4ac was indicated for the master regulators of SE, including *LEC* (*LEC1*, *LEC2* and *FUS3*) during TSA-induced SE. Moreover, we provided evidence on the role of HDA19 in controlling the *LEC2* expression in SE induction.

## 2. Materials and Methods

### 2.1. Plant Material

*Arabidopsis thaliana* (L.) Heynh. plants of the Columbia (Col-0) and Wassilewskija (Ws-2) WT ecotypes, the insertional mutant lines and the RNAi transgenic lines were used in the study (Tables S1 and S2). The seeds were purchased from the Nottingham Arabidopsis Stock Centre (NASC), Nottingham University, Nottingham, UK or were kindly provided by Dr. Kim Boutilier (Bioscience, Wageningen University and Research, Wageningen, Netherlands), Prof. Konstantinos Vlachonasios (School of Biology, Aristotle University of Thessaloniki, Thessaloniki, Greece) and Prof. Keqiang Wu (Institute of Plant Biology, National Taiwan University, Taipei, Taiwan). The homozygous mutants were selected according to the NASC standard protocol (http://signal.salk.edu/tdnaprimers.2.html; accessed on 28 February 2022).

### 2.2. Plant Growth and In Vitro Culture Conditions

The plants that were used as the source of the explants were grown in Jiffy-7 peat pots (Jiffy, Zwijndrecht, Netherlands) in a “walk-in” type phytotron under controlled conditions at 20–22 °C under a 16/8 h (light/dark) photoperiod of 100 µM m^−2^ s^−1^ white fluorescent light. The in vitro-grown plant material was then maintained at 20 °C under a 16/8 h (light/dark) photoperiod of 40 µM m^−2^ s^−1^ white fluorescent light.

### 2.3. In Vitro Culture of the Explants 

The explants, immature zygotic embryos (IZEs) at the cotyledonary stage of development, were cultured in vitro on different media or were immediately used as the control (0 d) for the molecular analysis. To obtain the explants for the in vitro cultures, the IZEs were manually isolated from the siliques under a stereomicroscope and transferred onto the culture media.

The basal E0 medium contained 3.2 g L^−1^ of B5 micro- and macro-elements (Duchefa Biochemie, Haarlem, Netherlands, #G0210), 20 g L^−1^ sucrose and 8 g L^−1^ agar, pH 5.8. For the SE induction, an EA medium based on the E0 medium that had been supplemented with 5.0 µM of 2,4-D (Duchefa Biochemie, Haarlem, Netherlands, #D0911) was applied following the standard protocol for SE induction in Arabidopsis [60]. Additionally, an E0 medium that had been supplemented with 1.0 µM of TSA (ET) (Sigma-Aldrich, St. Louis, MO, USA, #T1952) was also used.

The capacity for SE was evaluated in three-week-old cultures. Two parameters of embryogenic potential were evaluated: SE efficiency (the frequency of the explants that produced somatic embryos) and SE productivity (the average number of somatic embryos that developed per embryogenic explant). In order to evaluate SE efficiency and SE productivity, 10 explants were cultured in one Petri dish and 30 explants from each culture combination in at least three replicates were analysed.

The explants that were cultured on the E0, EA and ET media for 0, 5 and 10 days were sampled for molecular analysis using the different methods (Table S3).

### 2.4. Histone Extraction 

The histone proteins were isolated from the explants that had been cultured on the E0, EA and ET media for 0, 5 and 10 days using a commercially available kit according to the manufacturer’s protocol (Abcam, Cambridge, UK, #ab113476). The protein concentration was estimated using the Bradford assay with a Pierce Coomassie (Bradford) Protein Assay Kit (Thermo Fisher Scientific, Waltham, MA, USA, #23200) and the absorbance was measured using a Tecan Infinite M200 Microplate reader (Tecan, Männedorf, Switzerland) in a Bio-one Cellstar 96-well plate (Greiner, Kremsmünster, Austria) at a 595 nm wavelength. The samples were stored at −80 °C.

### 2.5. ELISA

The colourimetric ELISA method was used to analyse the total histone H3 acetylation in the different histone extracts, following the manufacturer’s protocol for a commercially available kit (Abcam, Cambridge, UK, #ab115124). The colourimetric absorbance was measured in a Tecan Infinite M200 Microplate reader at a 450 nm wavelength. The amount of acetylated H3 was calculated as the ng/mg protein. Wells without the antigen were used as the blank control. Three biological and two technical replicates of each culture combination were analysed for the total histone H3 acetylation content. 

### 2.6. ChIP-qPCR

The embryogenic culture of Arabidopsis was analysed using the ChIP method according to Nowak et al. [61]. A complex of proteins and DNA fragments were immunoprecipitated using the polyclonal antibodies against the acetylated forms of histone H3, H3K9/K14ac (2 µg; Sigma-Aldrich, St. Louis, MO, USA, #06-599) and histone H4, H4K5/K8/K12/K16ac (2 µg; Sigma-Aldrich, St. Louis, MO, USA, #06-866). For each sample, a negative control (mock) that had no antibody was analysed. The DNA that cross-linked to the immunoprecipitated proteins was reversed and 1 μL of ChIPed DNA was analysed with qPCR using the gene-specific primers (Table S4). The primers for qPCR for the genomic sequences that were located approximately 300 bp downstream of the transcription start site (TSS+300 bp) were designed in the Primer3Plus software [62]. The primers for *ACTIN7* (AT5G09810) were used according to Luo et al. [63]. A LightCycler 480 (Roche, Basel, Switzerland) real-time detection system was used to analyse the acetylation level of the chromatin that was associated with the analysed genes. The qPCR analysis followed Wójcikowska and Gaj [64]. The Ct values were calculated in LinRegPCR software [65]. The ChIP-qPCR data were normalised to the values that had been obtained for the internal control (*ACTIN7*) and the data are presented using the 2^ΔCt^ method where ΔCt represents the Ct *_ACTIN7_*-Ct_gene of interest_. Three biological replicates and two technical replicates were analysed.

### 2.7. Immunohistochemistry

The explants were fixed in 4% formaldehyde in PBS and placed in a vacuum desiccator for two hours. The procedures for embedding the tissue in Steedman’s wax and for preparing the slides were previously described by Wolny et al. [66]. The tissue was cut to 5 µm-thick tissue sections on a Zeiss Hyrax M40 rotary microtome (Zeiss, Oberkochen, Germany), placed on poly-L-lysine-coated slides and stretched by adding a small drop of water. The immunostaining was conducted as was described by Brąszewska-Zalewska et al. [67]. The polyclonal antibody against the acetylated form of histone H3 (H3K9/K14ac; 1:200; Sigma-Aldrich, St. Louis, MO, USA, #06-599) and Alexa Fluor 488 goat anti-rabbit IgG (1:200; Thermo Fisher Scientific, Waltham, MA, USA, #A-11008) as the secondary antibody were used. The slides were mounted and counterstained in Vectashield (Vector Laboratories, Burlingame, CA, USA, #H-1000) that contained 2.5 μg/mL DAPI. The DAPI fluorescence (excitation 405 nm, emission 425–475 nm) and Alexa 488 (excitation 488 nm, emission 500–600 nm) were registered using an Olympus FV1000 confocal system (Olympus, Tokyo, Japan) that was equipped with an Olympus IX81 inverted microscope. The images were processed using the ImageJ Fiji package. The fluorescence intensity of the Alexa 488 and DAPI was measured as the mean values from the Integrated Density (IntDen) parameter per the nuclei that represented the sum of all of the pixels within the region of interest. The eight-bit images with the Alexa 488 and DAPI fluorescence were segmented with the threshold value parameter. The results of these measurements were estimated in relative units as the mean values and 2–4 biological replicates were analysed per combination.

### 2.8. Reverse Transcription and RT-qPCR Analyses

An RNAqueous Total RNA Isolation Kit (Thermo Fisher Scientific, Waltham, MA, USA) was used to isolate the total RNA from the IZE explants that had been induced on the EA medium for 0, 5 and 10 days according to the manufacturer’s instructions. The concentration and purity of the RNA samples were evaluated using an ND-1000 spectrophotometer (NanoDrop Technologies, Wilmington, DE, USA). The RNA samples were treated with RQ1 RNase-free DNase I (Promega, Madison, WI, USA) according to the manufacturer’s instructions. The first-strand cDNA was synthesised using a RevertAid First Strand cDNA Synthesis Kit with an oligo-dT primer according to the manufacturer’s instructions (Thermo Fisher Scientific, Waltham, MA, USA). The obtained cDNA was diluted five-fold with water and used at a volume of 2.5 µL for the RT-qPCR, which was conducted according to Wójcikowska and Gaj [64]. A LightCycler 480 SYBR Green I Master (Roche, Basel, Switzerland) and the primers that were relevant to the genes being studied were used to determine the RT-qPCR reactions (Table S5). The Ct values were calculated in LinRegPCR software [65]. The relative gene expression levels were calculated and normalised to the internal control, the *TIN* (AT4G27090) gene, which encodes the 60S ribosomal protein [68]. The relative expression level was calculated using 2^−ΔΔCt^ where ΔΔCt represented ΔCt^reference condition^-ΔCt^compared condition^. Three biological replicates and two technical replicates were analysed.

### 2.9. RNA Isolation, Library Preparation and Sequencing

An RNAqueous Total RNA Isolation Kit (Thermo Fisher Scientific, Waltham, MA, USA) was used to isolate the total RNA from the IZE explants that had been induced on the different media (E0, EA, ET) for 0, 5 and 10 days according to the manufacturer’s instructions. Depending on the age of the culture, 250 (0 d), 200 (5 d) and 50 (10 d) explants were used to isolate the RNA. The concentration and purity of the RNA samples were evaluated using an ND-1000 spectrophotometer (NanoDrop Technologies, Wilmington, DE, USA). The integrity of the RNA was determined using an Agilent 2100 Bioanalyzer and Agilent RNA 6000 Nano chips (Agilent Technologies, Santa Clara, CA, USA). The RNA samples were treated with RNase-Free DNase and then purified with Acid-Phenol:Chloroform using the ammonium acetate method (Thermo Fisher Scientific, Waltham, MA, USA). The sequencing libraries were prepared using an Illumina ScriptSeq Complete Kit (Plant; Illumina, San Diego, CA, USA) following the manufacturer’s protocol. 2 µg of total RNA per sample were used as the input. Briefly, the library prep involved the following steps: removing the ribosomal RNA using Ribo-Zero rRNA Removal Reagents (Plant Leaf; Illumina, San Diego, CA, USA) followed by an ethanol precipitation of the rRNA-depleted sample, RNA fragmentation, cDNA synthesis, RNA removal, terminal tagging of the cDNA followed by a bead cleanup, PCR amplification using the Illumina indexes and a final bead purification. The quality of the prepared Illumina libraries was analysed using an Agilent Bioanalyzer with an Agilent High Sensitivity DNA Kit (Agilent Technologies, Santa Clara, CA, USA) and the quantities were estimated using a Qubit Fluorometer (Thermo Fisher Scientific, Waltham, MA, USA). To generate the clusters, the libraries were pooled at an equimolar concentration and sequenced using an Illumina HiSeq 4000 system (Illumina, San Diego, CA, USA) in the 2 × 76 cycles paired-end (PE) mode with six barcoded samples per lane.

### 2.10. RNA-seq Data Analysis

The sequencing data were processed in order to obtain fastq files with the bcl2fastq pipeline (Illumina, San Diego, CA, USA), including the demultiplexing and adapter trimming steps. The quality of the raw sequencing reads was evaluated with FastQC software (The Babraham Institute, Cambridge, United Kingdom) and all of the results were compared using the MultiQC tool [69]. As all of the reads were high quality, they were only soft-trimmed and filtered using Sickle [70]. Then, SortMeRNA was used to filter out any left-over fragments that had originated from the rRNAs [71]. The quality of the cleaned reads was assessed once again using FastQC and MultiQC. The cleaned reads were mapped to the *Arabidopsis thaliana* genome assembly GCA_000001735.1 (TAIR10) using the splice-aware aligner STAR [72] with the mapping parameters adjusted to the Arabidopsis genome characteristics, a basic two-pass mode and in order to permit 5% of mismatches to the reference genome. The unique counts per gene were calculated in a built-in option in STAR and were used to analyse any further differential gene expression. The mapping quality was assessed using the SAMStat package [73] and Qualimap [74]. The sequence alignment files were indexed using SAMtools [75] and the mapped reads were visually inspected using an Integrative Genomics Viewer [76]. All of the further computational and graphical analyses were performed in the *R* environment. The size factors of the samples were estimated using the median ratio method and the counts were normalised using the DESeq2 algorithm [77]. To inspect and visualise the data, the counts were regularised and log-transformed (rlog) in order to obtain the log2-scaled data that were approximately homoscedastic and normalised with respect to the library size. The differential expression was analysed with DESeq2 software [77], assuming a negative binomial distribution and using a general linearised model with prior beta shrinkage. Wald’s exact test was used to identify the differentially expressed genes (DEGs) under the *p*-value adjustment (*p* < 0.05) for multiple comparisons using the Benjamini-Hochberg False Discovery Rate (FDR) correction [78].

### 2.11. Statistical Analysis 

The Student’s *t*-test (*p* < 0.05) or two-way ANOVA analysis (*p* < 0.05) followed by Tukey’s HSD test (*p* < 0.05) were used to determine any significant differences between the compared combinations. The graphs show the means with the standard deviations (SD).

## 3. Results

### 3.1. An Increased H3ac Level Accompanies SE Induction 

In Arabidopsis, auxin treatment is required to induce SE in in vitro-cultured explants. However, we previously indicated that TSA treatment also resulted in SE induction on an auxin-free medium, which suggests that Hac plays a role in the mechanism that controls the embryogenic reprogramming of plant somatic cells [38]. To verify this hypothesis, we analysed the global H3ac level in Arabidopsis explants that had been induced toward SE on auxin (2,4-D)- and TSA-supplemented media, EA and ET, respectively. The control combinations included freshly isolated, non-induced explants (0 d) and explants that had been cultured on a non-embryogenic E0 medium.

**Figure 1:**
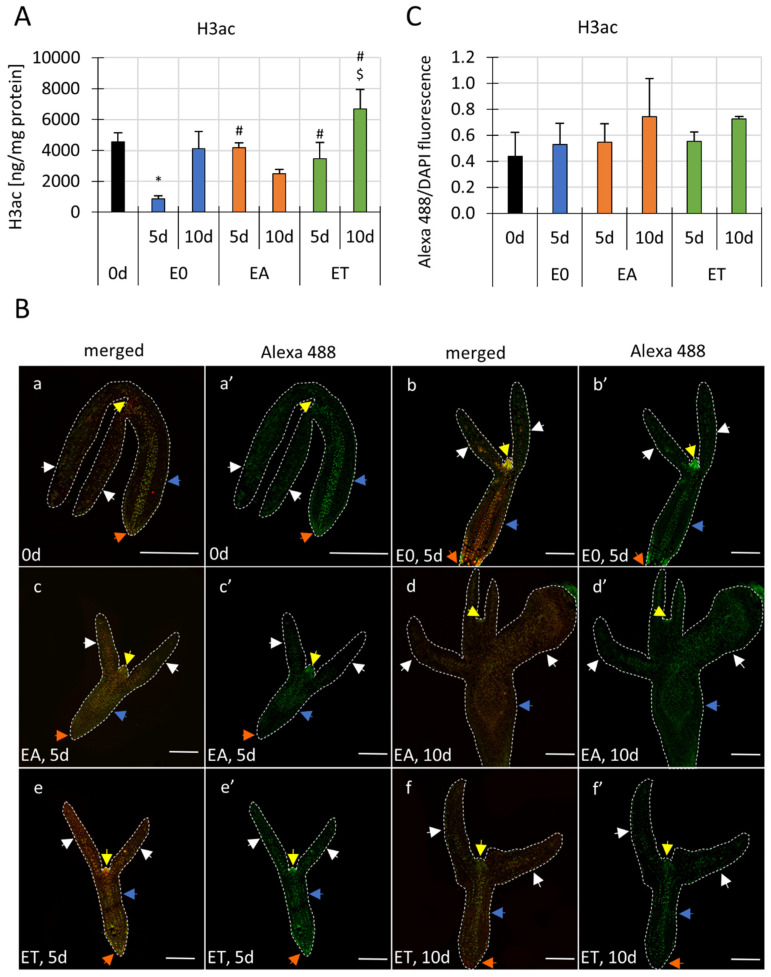
Analysis of H3a in SE. (**A**) Total histone H3 acetylation (H3ac) level in the freshly isolated and in vitro-cultured explants of Col-0. The explants were induced on E0, EA and ET media for 5 and 10 days. The amount of the acetylated form of histone H3 was calculated as the ng/mg of protein. (**B**) Immunodetected spatio-temporal pattern of Hac in the Col-0 explants and immature zygotic embryos that had been cultured on different media (EA, ET, E0). H3ac-signals in longitudinal sections of the freshly isolated 0 d explants (a, a’) and the explants that had been cultured for 5 and 10 days on different media, including E0 (b, b’), EA (c, c’, d, d’) and ET (e, e’, f, f’). Coloured arrows point to the cotyledon (white), SAM (yellow), RAM (orange) and hypocotyl (blue). Red (computer altered)—DAPI; green (computer altered)—Alexa 488 (immunostaining of H3ac). Scale bar represents 200 µm. (**C**) The intensity of Alexa 488 (H3ac) fluorescence in the cell nuclei of the explant cotyledons was normalised to the fluorescence of DAPI (DNA) and data are presented as the Alexa 488/DAPI ratio. (**A**,**C**) A two-way ANOVA analysis (*p* < 0.05) followed by Tukey’s HSD (*p* < 0.05) was used to determine any values that were significantly different from 0 d (∗); the E0 culture at the same age (#); the EA culture at the same age ($) (n = 2–4; means ± SD are given).

The results of the ELISA test indicated significant differences in the global H3ac level between the control (E0) and embryogenic (ET, EA) cultures (Figure 1A). We found that the non-induced explants that had been cultured on the E0 medium had a transient (5 d) reduction in global H3 acetylation. In contrast, no deacetylation of histones was observed in the SE explants that had been induced on the EA and ET media. In addition, the H3ac level was higher in the ET- than in the EA-induced explants.

Altogether, the results regarding the global changes in the H3 acetylation levels in the SE-induced explants suggested that both SE-induction treatments, auxin and TSA, affected the Hac level in the cultured explants. 

### 3.2. The Differential Spatio-Temporal Histone H3ac Pattern in the SE-Induced Explants 

Next, the immunohistochemical approach was used to examine the spatio-temporal H3ac pattern during SE. To obtain a general view of the Hac pattern in the in vitro-cultured explants, we examined the distribution of H3ac in the freshly isolated (0 d) and in the in vitro-cultured explants (EA, ET and E0). In the control E0 culture, the analysis was limited to the fifth day because the explants that had been cultured for 10 days had developed into seedlings. 

The results indicated that there were H3ac signals in different explant parts, including in the cotyledons, SAM and hypocotyls (Figure 1B). Some differences in the general distribution pattern of Hac fluorescence were observed between the control and the SE-induced explants. In the freshly isolated 0 d explants, strong fluorescence signals were concentrated along with the vascular tissue in the hypocotyl (Figure 1B; a–a’) while at 5 d of E0 culture, a strong Hac was detected in the SAM area (Figure 1B; b–b’). Strong SAM-associated Hac signals were also observed in the SE-induced explants, particularly in the early 5 d culture (Figure 1B; c–c’, e–e’). Unlike the E0, the SE-induced explants had signals that were more dispersed in the whole explant, which were distributed evenly in the hypocotyl and cotyledons. Therefore, we assumed that the explant parts with intensively enriched Hac signals, including the hypocotyls and SAM, primarily contributed to changes in the global H3ac that was detected in the ELISA assay (Figure 1A). 

In the SE-induced IZE explants of Arabidopsis, somatic embryos are produced exclusively on the adaxial side of the cotyledons in close proximity to the SAM, while the rest of the explant tissue, including the hypocotyl, SAM and RAM (root apical meristem) are not involved in SE [79]. Therefore, we gained a better insight into the SE-involved cotyledons and we compared the intensity of the Hac signals between the treatments in the selected areas of the tissue (Figure S1). The results indicated that there were no significant differences in the H3ac fluorescence signals in the SE-involved cotyledonary tissue between the compared combinations (Table S6, Figure 1C). 

Taken together, the results of the Hac immunohistochemical analysis indicated a spatio-temporal modulation of the H3ac signal in both the embryogenic and non-embryogenic cultures. In the SE-induced explants, the intense H3ac signals did not specifically colocalise with the SE-involved explant parts, i.e., the cotyledons. Moreover, the SE-induced cotyledons had levels that were similar to the control H3ac. 

### 3.3. Changes in H3 and H4 Acetylation in Gene-Bound Chromatin Are Associated with the Differential Transcription of the Key SE-Involved TF Genes in TSA-Induced SE

Given that the *TF* genes with an SE-regulatory function are deregulated in the TSA-induced SE [37,38], we assumed that Hac might contribute to SE induction by controlling these genes. To address this critical issue, we analysed the relationship between the Hac and the expression levels of the SE-involved *TF*s, including *LEC1*, *LEC2*, *FUS3*, *MYB118*, *BBM*, *AGL15* and *WUS* in the embryogenic (ET, EA) vs. the control (E0) cultures. Using the ChIP-qPCR method, we evaluated the H3ac and H4ac enrichment in the chromatin that is associated with the TSS+300 bp fragments of the analysed genes. The effect of the auxin and TSA treatments on Hac and gene expression relative to the control E0 culture was evaluated. 

**Figure 2:**
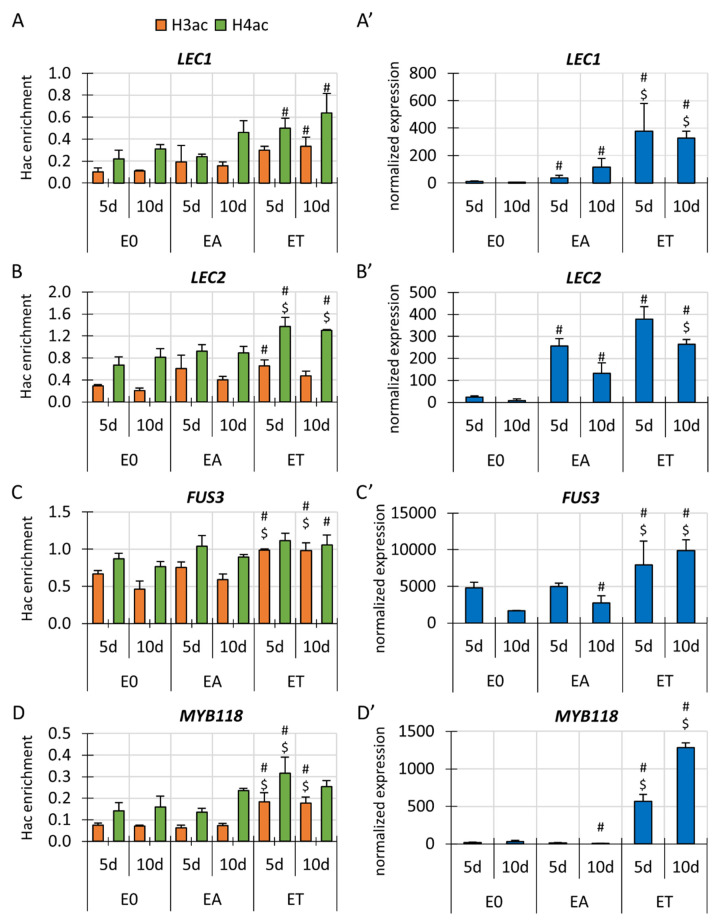
The H3ac and H4ac levels and expression profiles of *LEC1* (**A**,**A’**), *LEC2* (**B**,**B’**), *FUS3* (**C**,**C’**) and *MYB118* (**D**,**D’**) in the Col-0 explants that had been cultured on the E0, EA and ET media for 5 and 10 days. Hac enrichment indicates the amount of DNA after ChIP that was normalised to the internal control (*ACTIN7*). ChIP-qPCR: a two-way ANOVA analysis (*p* < 0.05) followed by Tukey’s HSD test (*p* < 0.05) were used to determine the values that were significantly different from the E0 culture at the same age (#); the EA culture at the same age ($) (n = 3; means are given ± SD). RNA-seq: Wald’s exact test was used to identify any differentially expressed genes (DEGs) under the *p*-value adjustment (*p* < 0.05) for multiple comparisons with the Benjamini-Hochberg False Discovery Rate (FDR) correction. Values that were significantly different from the E0 culture at the same age (#); the EA culture at the same age ($) (n = 3; means are given ± SD).

The analysis indicated a significant increase in Hac in the chromatin that is associated with four of the analysed *TF* genes, *LEC1*, *LEC2*, *FUS3* and *MYB118* in the TSA-induced culture (Figure 2A–D). Consistent with this result, the TSA treatment also positively affected the expression level of the *LEC1*, *LEC2*, *FUS3* and *MYB118* genes, thus implying a positive relationship between Hac and the expression level of these genes (Figure 2A’–D’). In contrast, the three other *TF* genes, *BBM*, *AGL15* and *WUS*, which had a significantly increased expression in the embryogenic cultures (Figure S2A’–C’), had similar H3ac and H4ac levels in both the embryogenic and non-embryogenic cultures (EA vs. E0; ET vs. E0) (Figure S2A–C).

Altogether, the result provided evidence on the role of Hac in controlling the *TF* genes with a regulatory function in the embryogenic response, including *LEC1*, *LEC2*, *FUS3* and *MYB118* during TSA-induced SE. 

### 3.4. Different Genes Encoding the Histone Acetyltransferase (HATs) and Deacetylases (HDACs) Are Involved in SE Induction

The results regarding the changes in Hac in SE induction suggested the role of histone acetyltransferases (HATs) and deacetylases (HDACs) in controlling the SE transcriptome. Therefore, in order to identify the specific histone acetyltransferases and histone deacetylases that control SE induction, the expression patterns of the *HAT* and *HDAC* genes that are encoded in the Arabidopsis genome were analysed in the explants that had been induced on the different media (ET, EA and E0). Following the results regarding the *HAT/HDACs* profiling, the embryogenic capacity of the relevant *hat* and *hdac* mutants was evaluated.

#### 3.4.1. *HAT* Genes 

**Figure 3:**
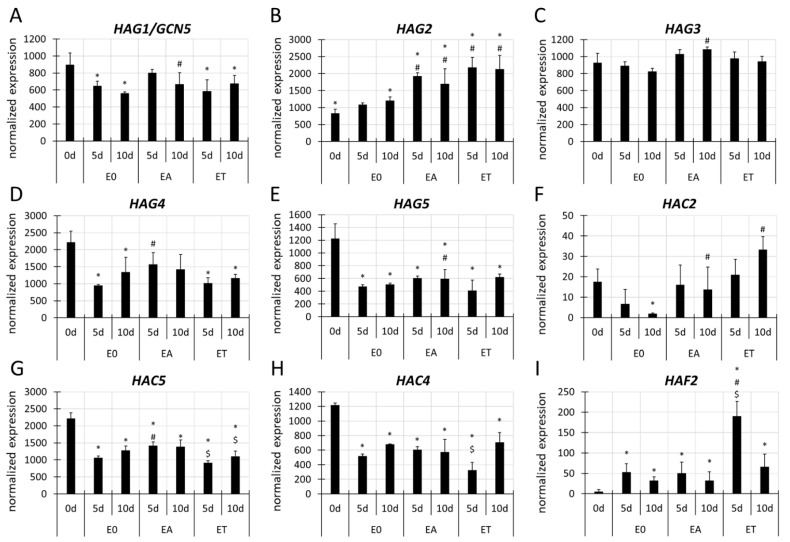
Differential expression of the *HAT* genes, including *HAG1/GCN5* (**A**), *HAG2* (**B**), *HAG3* (**C**), *HAG4* (**D**), *HAG5* (**E**), *HAC2* (**F**), *HAC5* (**G**), *HAC4* (**H**) and *HAF2* (**I**), in the Col-0 explants that had been cultured on the E0, EA and ET media for 0, 5 and 10 days. Graphs represent the data from the RNA-seq analysis. Wald’s exact test was used to identify any differentially expressed genes (DEGs) under the *p*-value adjustment (*p* < 0.05) for multiple comparisons with the Benjamini-Hochberg False Discovery Rate (FDR) correction. Values that were significantly different from 0 d (∗); the E0 culture at the same age (#); the EA culture at the same age ($) (n = 3; means ± SD are given).

The RNA-seq data on the expression of the 12 *HAT*s that are encoded in the Arabidopsis genome [80] indicated that SE induction is associated with the upregulation of numerous genes that represent different gene families, including *GNAT, MYST*, *CBP* and *TAF_II_250* (Table S7, Figure 3). Auxin treatment resulted in an increased expression (FC 1.3–5.9) of seven *HAT* (*HAG1/GCN5*, *HAG2*, *HAG3*, *HAG4*, *HAG5*, *HAC2* and *HAC5*) genes (Figure 3A–G), while three genes, *HAG2*, *HAC2* and *HAF2* (Figure 3B,F,I) were upregulated in the TSA-induced culture compared to the E0 culture. Two *HAT*s, *HAG2* and *HAC2* (Figure 3B,F), were upregulated in response to both the EA and ET media. In particular, *HAC2* was highly responsive to both treatments and had a 5.9 and 12.6 FC increase in its transcript level in the EA and ET culture, respectively (Figure 3F). In contrast, *HAC4* was downregulated in the TSA-induced culture compared to the EA culture (Figure 3H). Together, the results imply the activation of different *HAT* genes in SE induction and the role of auxin and Hac in regulating these genes might be assumed.

**Figure 4:**
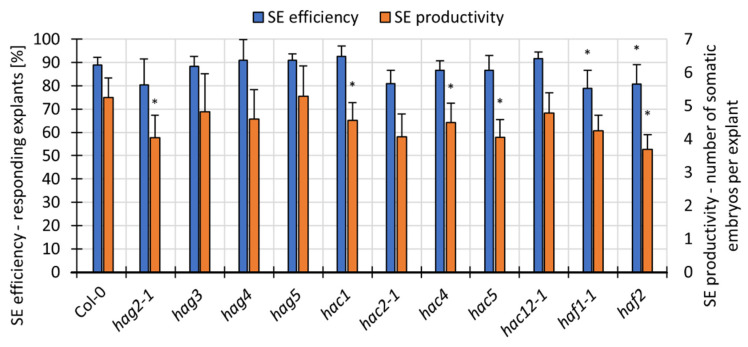
The embryogenic capacity of the *hat* mutants on the EA medium evaluated in the 21-day-old culture. Values significantly different from the control WT culture (Col-0) are marked with an asterisk (n ≥ 4; means ± SD are given) (Student’s *t*-test, *p* < 0.05).

Next, in order to identify the specific *HAT*s that are involved in auxin-induced SE, we analysed the embryogenic response of different *hat* mutants in a standard embryogenic culture on an EA medium. The results indicated a defective SE response of six *hat* mutants (*hag2*, *hac1*, *hac4*, *hac5, haf1 and haf2*); their explants produced somatic embryos with a distinctly decreased efficiency or productivity in response to the auxin treatment (Figure 4). It is worth noting that the defective SE-response of four mutants, *hac1, hac4, hac5* and *haf2,* was specific to the EA culture because the embryogenic response of these mutants was not impaired on the ET medium (Figure S3). This finding suggests that the function of the relevant *HAT*s, *HAC1*, *4*, *5* and *HAF2* genes might be specific to auxin-induced SE. 

**Figure 5:**
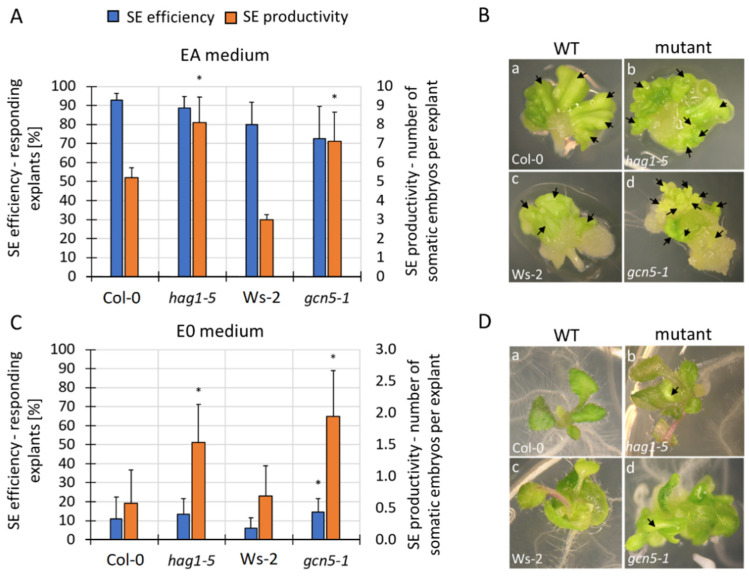
The increased embryogenic capacity of the *hag1/gcn5* mutants in the explants that had been cultured for 21 days on the EA (**A**,**B**) and E0 (**C**,**D**) medium. Arrows indicate the somatic embryos (**B**) and embryo-like structures (**D**). Values significantly different from the control WT culture (Col-0 or Ws-2) are marked with an asterisk (**A**,**C**) (n ≥ 6; means ± SD are given) (Student’s *t*-test, *p* < 0.05).

In contrast to the numerous *hat* mutants with SE-impaired potential, mutations in the *HAG1/GCN5* gene resulted in an improved embryogenic response (Figure 5). In response to the EA treatment, the *hag1–5* and *gcn5–1* mutant explants produced a higher average number of somatic embryos per explant (Figure 5A,B). In addition, the *hag1–5* and *gcn5–1* explants were also able to induce a somatic embryo on the control E0 medium (Figure 5C,D). Therefore, a negative role of HAG1/GCN5 in auxin-mediated SE might be implied.

To summarise, the results imply a role of the genes encoding different HATs in the embryogenic transition of explant cells. Most of the SE-involved *HAT*s, including *HAG2, HAC1, HAC4, HAC5, HAF1* and *HAF2*, seem to positively contribute to SE induction while *HAG1/GCN5* might control this process negatively. 

#### 3.4.2. *HDAC* Genes

**Figure 6:**
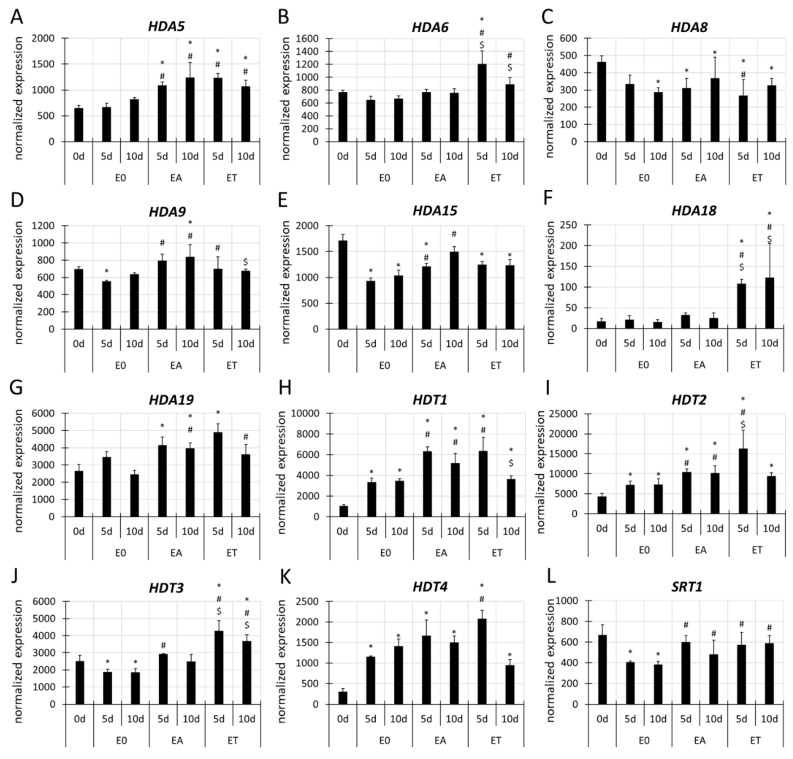
Differential expression of the *HDAC* genes, including *HDA5* (**A**), *HDA6* (**B**), *HDA8* (**C**), *HDA9* (**D**), *HDA15* (**E**), *HDA18* (**F**), *HDA19* (**G**), *HDT1* (**H**), *HDT2* (**I**), *HDT3* (**J**), *HDT4* (**K**) and *SRT1* (**L**) in the Col-0 explants that had been cultured on the E0, EA and ET media for 0, 5 and 10 days. Graphs represent the data from the RNA-seq analysis. Wald’s exact test was used to identify any differentially expressed genes (DEGs) under the *p*-value adjustment (*p* < 0.05) for multiple comparisons with the Benjamini-Hochberg False Discovery Rate (FDR) correction. Values that were significantly different from 0 d (∗); the E0 culture at the same age (#); the EA culture at the same age ($) (n = 3; means ± SD are given).

In Arabidopsis, 18 *HDAC* genes, which are grouped into three gene families, *RPD3/HDA1*, *SIR2* and plant-specific *HD2*, encode the histone deacetylases [80]. The RNA-seq data on the embryogenic (EA, ET) vs. control (E0) cultures revealed an increased expression of the majority (12/18) of the *HDAC*s in response to the embryogenic treatments (Table S8, Figure 6). Seven SE-upregulated *HDAC*s had an increased expression in the EA and ET cultures, including *HDA5*, *HDA9, HDA19*, *HDT1*, *HDT2*, *HDT3* and *SRT1* (Figure 6A,D,G–J,L). Four *HDAC*s had a treatment-specific upregulation, including the *HDA15* (Figure 6E) in EA and *HDA6*, *HDA18* and *HDT4* (Figure 6B,F,K) in the ET culture. In addition, *HDA8* was downregulated in the TSA-induced culture compared to the E0 culture (Figure 6C). Therefore, we assumed that although both the auxin and TSA treatments seemed to positively regulate most of the *HDAC* genes, the contribution of individual genes to TSA- vs. auxin-induced SE might differ. 

Altogether, the results indicated that the substantial upregulation of numerous *HDAC* genes in SE induction seems to be controlled by auxin and Hac.

**Figure 7:**
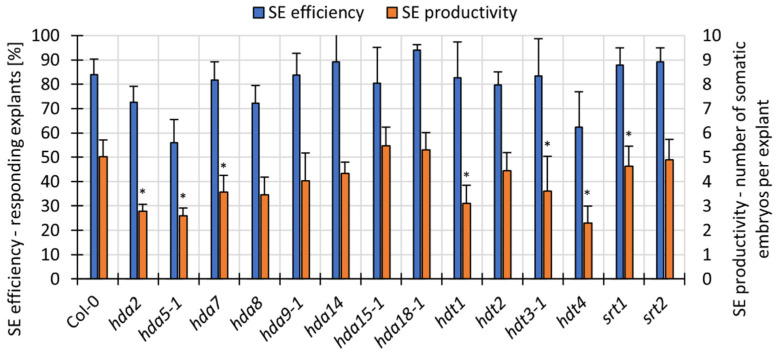
The embryogenic capacity of the *hdac* mutants on the EA medium evaluated in the 21-day-old culture. Values that were significantly different from the control WT culture (Col-0) are marked with an asterisk (n ≥ 4; means ± SD are given) (Student’s *t*-test, *p* < 0.05).

**Figure 8:**
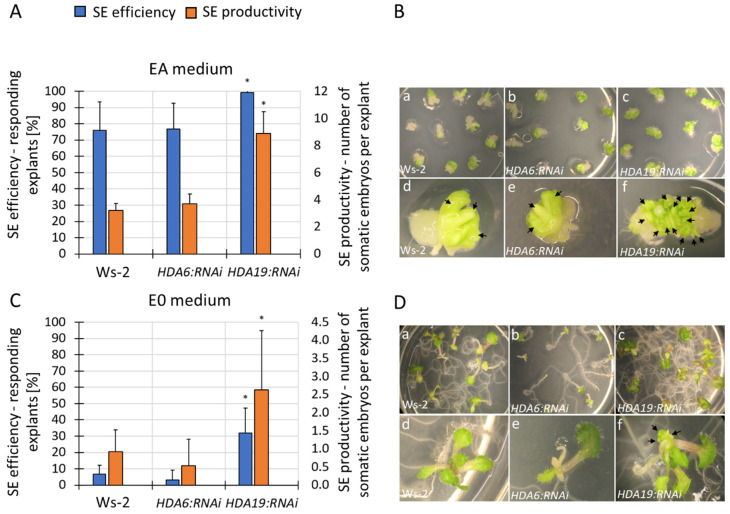
The embryogenic capacity of the *HDA6:RNAi* and *HDA19:RNAi* explants that had been cultured on the EA (**A**,**B**) and E0 (**C**,**D**) media for 21 days. Values that were significantly different from the control WT culture (Ws-2) are marked with an asterisk (**A**,**C**) (n ≥ 5; means ± SD are given) (Student’s *t*-test, *p* < 0.05). Arrows indicate the somatic embryos (**B**: d, e, f) and embryo-like structures (**D**: f).

To gain further insight into the specific HDACs that are involved in auxin-induced SE, we analysed the *hdac* mutants and transgenic RNAi lines in terms of their capacity for an embryogenic response. The analysis indicated seven mutants that had a significantly lower SE productivity, including *hda2*, *hda5*, *hda7*, *hdt1*, *hdt3*, *hdt4* and *srt1* in the EA vs. E0 cultures (Figure 7). In contrast, the *HDA19:RNAi* explants had an improved embryogenic response, which was manifested by the distinctly higher number of somatic embryos that were produced on the EA medium (Figure 8A,B). Moreover, this line also produced somatic embryos on the control auxin-free E0 medium (Figure 8C,D). The improved embryogenic potential of the *HDA19:RNAi* culture suggests that there is a negative impact of the *HDA19* gene on SE induction.

Altogether, the gene expression profiling and mutant analysis results imply that different *HAT*s and *HDAC*s might positively or negatively contribute to SE induction. Most of the SE-deregulated *HAT*s and *HDAC*s, including *HDA2, HDA5, HDA7, HDT1, HDT3, HDT4* and *SRT1*, might positively control SE induction. In contrast, *HAG1/GCN5* and *HDA19* seem to negatively regulate SE response.

### 3.5. Mutations in HAT and HDACs Affect the Expression of Essential SE-TF Genes

**Figure 9:**
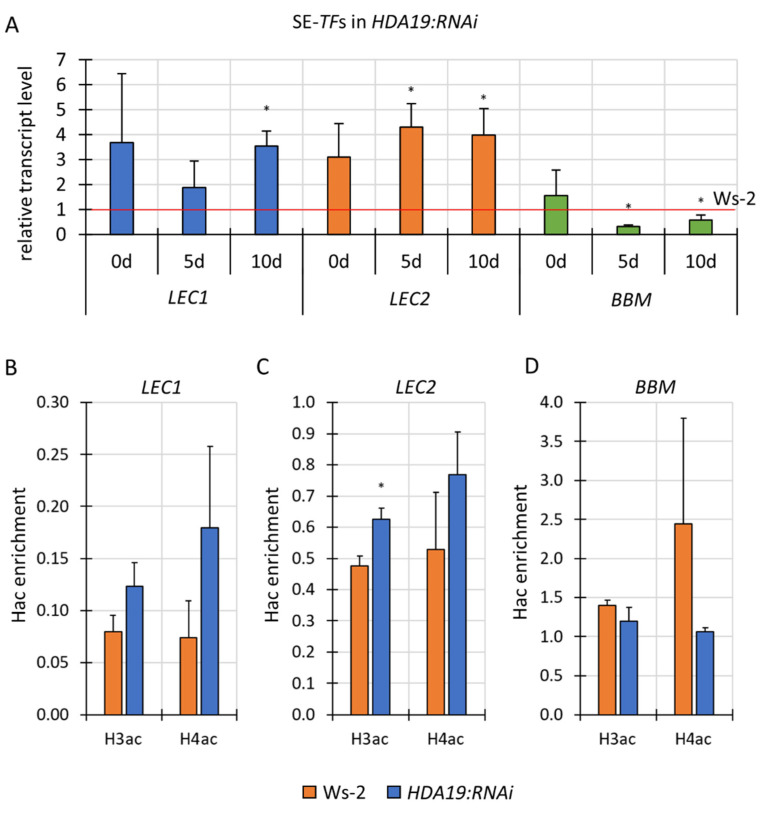
The involvement of HDA19 in controlling the *LEC1, LEC2* and *BBM* genes in SE induction. Expression profiles of the SE-*TF*s in freshly isolated (0 d) and explants that had been cultured on the EA medium (5 and 10 d) of the *HDA19:RNAi* line (**A**). The relative transcript level was normalised to the internal control (*TIN* gene) and calibrated to the control WT cultures of Ws-2. Graph represents the data from the RT-qPCR analysis. The H3ac and H4ac levels in the chromatin that is associated with *LEC1* (**B**) *LEC2* (**C**) and *BBM* (**D**) in SE. The Hac levels in the chromatin that is bound to the TSS+300 bp gene fragment were evaluated in the *HDA19:RNAi* and Ws-2 (WT) explants that had been cultured on the EA medium for five days. The Hac enrichment indicated the amount of DNA after ChIP that was normalised to the internal control (*ACTIN7*). An asterisk indicates values that were significantly different than the ones observed in Ws-2 (n = 3; means ± SD are given) (Student’s *t*-test, *p* < 0.05).

For further insights into the mechanisms by which HATs and HDACs might contribute to the embryogenic response, we analysed the expression of the *LEC1, LEC2* and *BBM* genes in the EA-induced explants of the *hat* (*hag1/gcn5*) and *hdac* (*hdt1, hdt4, HDA19:RNAi)* lines that were significantly affected SE response (Figure 5, Figure 7 and Figure 8). The results indicated substantial changes in the expression level of the SE-*TF*s in the mutant cultures. The diversity of the gene expression profiles between the genes was indicated and both an up- and downregulation of the genes were observed. The silencing of *HDA19* affected different SE-*TF*s differently. A significant downregulation of *BBM* (FC 0.3–0.6) and upregulation (FC 3.5–4.3) of *LEC1* and *LEC2* were observed in the explants (0 d) and the SE culture of *HDA19:RNAi* (Figure 9A). The results suggest that HDA19 might regulate *LEC1* and *LEC2* negatively while it impacts *BBM* positively. 

Gene profiling in the other two *hdac* mutants, *hdt1* and *hdt4*, revealed a significantly decreased expression of all three genes, *LEC1*, *LEC2* and *BBM* (FC 0.02–0.2), in the explants (0 d) and during SE culture (Figure S4A). The results imply a positive impact of HDT1 and HDT4 on the SE-*TFs* expression. In contrast, histone acetyltransferase HAG1/GCN5 seems to affect the SE-*TF* genes differently. The gene expression profiling of the *gcn5-1* mutant explants revealed an increased expression level of *LEC1* (FC 3.0–5.5) and *LEC2* (FC 1.2–3.6) and the downregulation of *BBM* (FC 0.2–0.7) in the freshly isolated (0 d) and EA-induced explants (Figure S4B). These results suggest that HAG1/GCN5 might negatively control *LEC1* and *LEC2* while positively regulating *BBM* during auxin-induced SE.

Taken together, the results regarding gene profiling in the *hat/hdac* mutant cultures provide some suggestions that HAT and HDACs by fine-tuning the *TF* genes, including *LEC1*, *LEC2* and *BBM*, might control the embryogenic response. However, the diverse expression profiles of the SE-*TF*s in the *hat* and *hdac* mutants imply that further analyses are required to reveal the complex and gene-specific mechanisms by which different HAT/HDACs might affect the SE-*TF*s during embryogenic induction.

### 3.6. HDA19 Might Negatively Control LEC2 in SE Induction via Histone Deacetylation

The improved embryogenic response (Figure 8), together with modulated *TF* gene expression in the *HDA19:RNAi* culture (Figure 9A), motivated us to investigate whether HDA19 might control the SE-*TF*s via histone acetylation. To answer this question, we analysed the Hac level in the chromatin that is associated with *LEC1*, *LEC2* and *BBM* in the *HDA19:RNAi* and WT (Ws-2) explants that had been cultured on the EA medium (Figure 9B–D). ChIP-qPCR indicated a significant H3ac enrichment in the chromatin that was associated with *LEC2* in the *HDA19:RNAi* culture (Figure 9C). This result, together with the increased *LEC2* transcription in the *HDA19:RNAi* culture (Figure 9A), supports the assumption of a role of HDA19 in the negative control of *LEC2* in SE induction.

In contrast to *LEC2*, the Hac level in the chromatin that is associated with the TSS+300 bp fragment of the *LEC1* and *BBM* genes was not significantly affected in the *HDA19:RNAi* culture (Figure 9B,D). The results indicated a lack of any Hac changes in the chromatin that is bound to the TSS+300 bp regions of the *LEC1* and *BBM* genes in response to the silencing of *HDA19*. The mechanism of the HDA19-mediated control of these genes during auxin-induced embryogenic response requires further analysis.

## 4. Discussion

### 4.1. Changes in Histone Acetylation Accompany SE Induction

The developmental reprogramming of somatic cells in animals and plants is associated with extensive changes in the cell transcriptome that is controlled by the compactness of chromatin [10,81,82]. Accordingly, histone acetylation-mediated chromatin accessibility has been postulated to control genes in the in vitro-induced plant development, including the embryogenic transition of the somatic cells that accompany SE induction [16,83]. 

These results indicated distinct differences in the Hac abundance in the embryogenic vs. non-embryogenic cultures of Arabidopsis (Figure 1). A substantial decrease in the global H3 acetylation level was specific to the non-embryogenic control culture. Stress factors have been widely documented as distinctly affecting Hac in plants [84,85]. Therefore, we assumed that the stress that is imposed by in vitro-culture conditions would negatively affect Hac in the explant tissue. In support of this, the SE-induced explants had a significantly higher level of global H3ac than the non-embryogenic culture. Hence, we hypothesised that TSA and auxin seem to counteract the in vitro-culture/stress-induced histone deacetylation, thereby increasing Hac and inducing SE in the treated explants.

In line with the increased Hac level in the ET culture, histone hyperacetylation after TSA treatment has been reported in different plant developmental processes in vivo [86] and in vitro [28,87]. The TSA-increased Hac is believed to result from the inhibition of the HDACs of the RPD3/HDA1 and HD2-type families, which are the targets of TSA [88,89]. Similar to TSA, a positive effect of auxin treatment, including 2,4-D, on the Hac level in in vitro-cultured cells and tissue has also been reported [25,90,91]. Consistent with the auxin-induced changes in the Hac level, the involvement of Hac in the auxin-induced transcriptional regulation of genes has also been demonstrated [92,93,94]. In this mechanism, auxin affects the recruitment of HDACs and HATs to the transcriptional complexes that differentially acetylate histones at the target loci [95].

The immunohistochemical analysis of the Hac pattern in the IZE explants showed that the meristematic zones (SAM and RAM) and vascular tissue in the hypocotyls of the zygotic embryos, both those that were freshly isolated and those that were in vitro cultured had the most intense Hac signals. Similarly, the Hac signals were co-localised with the meristematic regions in the zygotic embryos of *Hordeum vulgare* and *Brachypodium distachyon* [66,96]. Enhanced H4ac signals have also been reported in the meristematic regions of microspore-derived *B. napus* embryos as well as the vegetative and floral buds of plants [31,97,98]. Different cell types, including meristematic cells, might exhibit specific patterns of epigenetic modifications as was indicated for DNA methylation in the RAM and histone methylation in the SAM of Arabidopsis [99,100]. Whether the higher accumulation of Hac might be attributed to cells with a meristematic identity remains to be verified. 

It is worth noting that the pattern of the Hac signals in the explants seems to coincide with the IAA accumulation regions in a zygotic embryo that involve the meristematic (SAM, RAM) and vascular tissues [101,102]. However, less intensive, dispersed patches of Hac signals were also found in the IZE cotyledons that are directly involved in the development of the somatic embryos [79]. Moreover, auxin biosynthesis followed by an increase in IAA in SE-involved explant tissue has also been postulated [45,56,103]. In support of the postulated convergence between Hac and the IAA accumulation, the role of Hac in auxin biosynthesis, including the regulation of the *YUCCA* genes that control IAA biosynthesis in SE, has been indicated [45,104,105]. Thus, we hypothesised that the Hac signals in the cotyledons, similar to the meristematic and vascular tissues, might co-localise with the areas with an increased IAA content that are engaged in somatic embryo development. An analysis of the Hac vs. IAA accumulation in the distinct SE-involved sites of the cotyledons might verify the functional relevance of Hac and auxin in the embryogenic reprogramming of explant cells.

The immunohistochemical assay revealed global changes in the spatio-temporal Hac pattern in the in vitro-cultured explants, both the control and SE-induced explants. Hac signals were detected in different explant parts and they were not specifically co-localised with the SE-involved cotyledons. Moreover, the quantification of the H3ac signals in the cotyledons exhibited no significant differences between the embryogenic (EA and ET) and control (E0) cultures. A possible explanation for this result provided a recent report on rather limited heterochromatin decondensation in the SE-involved cotyledon cells in response to auxin [106]. Together, these results imply that discrete and cell-specific changes in Hac might accompany SE induction. That implies that revealing the discrete Hac-related changes in chromatin of SE-induced explants might require an analysis of specific auxin-responsive protodermal cells at the adaxial side of cotyledons [79]. 

### 4.2. Hac Controls LEC1, LEC2 and BBM during TSA-Induced Embryogenic Reprogramming

The results regarding the global changes in Hac abundance in the SE-induced explants implied the role of Hac in the embryogenic reprogramming of somatic cells. To reveal the regulatory mechanism by which Hac controls SE, we analysed the changes in the Hac in the chromatin that is associated with the *TF* genes that have regulatory functions in embryogenic induction. The Hac-regulated candidates involved the *TF* genes with a TSA-affected expression, including *LEC1*, *LEC2*, *FUS3*, *BBM*, *AGL15* and *MYB118* [37,38,87]. The results of the ChIP-qPCR indicated that the increased transcription of *LEC1, LEC2, FUS3* and *MYB118* in the TSA-induced SE was associated with an accumulation of H3ac and H4ac in the gene-bound chromatin (Figure 2). The analysed H3 (H3K9ac and H3K14ac) and H4 (H4K5ac, H4K8ac, H4K12ac and H4K16ac) lysine residues activate gene transcription in plants and they are frequently enriched in the TSS regions of genes [107]. The reported role of H3/H4ac marks in the regulation of different genes [4,108] together with a significant increase in the H3 and H4 acetylation and transcript abundance of the *LEC1*, *LEC2,* and *FUS3* imply the role of Hac in the transcriptional control of these genes during TSA-induced SE (Figure 2). Congruently, the role of Hac in regulating *LEC1* and *LEC2* in Arabidopsis during seedling development was reported [57,58,59]. The results of the ChIP-qPCR indicated that in contrast to TSA, the explants that had been treated with auxin had no significant modulation of the H3ac and H4ac levels in the analysed chromatin fragments that were bound to the SE-*TF*s. The results suggest that different gene regions and/or lysine marks in the SE-*TF*s-bound chromatin might be targeted by auxin and TSA in the treated explants. 

Consistent with this assumption, the chromatin that was bound to TSS gene regions other than those analysed, including the promoters of the SE-*TF*s might undergo a differential Hac in response to auxin [94]. The lysines that are targeted by auxin-mediated histone acetylation include the H3K27 mark [93,94] which was not analysed in the present study. Thus, an analysis of other lysine marks, predominantly H3K27ac, in the different regions of the SE-*TF*s might be important in studies on the Hac-related mechanisms that control the genes during auxin-induced SE. The present results show that a complex and to date only partially recognised mechanism of the auxin-controlled Hac [95] might be different from the one that is involved in TSA-induced Hac in which the inhibition of the HDACs promotes Hac [85]. Further analysis is required to reveal the common and treatment-specific elements of the auxin- vs. TSA-triggered gene regulation via Hac.

### 4.3. Numerous HATs and HDACs Control SE Induction

In SE induction, the changes in Hac result from the activity of the antagonistically acting enzymes, HATs and HDACs, that play a role in regulating the genes in plant development [109,110]. In support of the involvement of HAT/HDACs in SE induction, a differential expression of the *HAT/HDAC* genes and the modulated activity of the encoded enzymes have been reported in the embryogenic cultures of different plants, including Arabidopsis [30,38,111]. Similarly, we revealed the deregulation of several *HAT* (*HAG1/GCN5*, *HAG2, HAG3, HAG4, HAG5, HAC2, HAC4, HAC5* and *HAF2*) and *HDAC* (*HDA5*, *HDA6, HDA8,* *HDA9, HDA15, HDA18, HDA19*, *HDT1*, *HDT2*, *HDT3, HDT4* and *SRT1*) genes in the 2,4-D- and TSA-induced embryogenic explants (Figure 3 and Figure 6). The differential expression of *HAT*s and *HDAC*s in response to the HDAC-inhibitors and auxin treatment has also been indicated by others, which suggests that Hac and auxin play a role in the transcriptional control of these genes [36,112,113,114]. Accordingly, Hac has been indicated in the chromatin that is associated with the *HAT/HDACs* genes (PlantPan 3.0, [115]). In support of an auxin-responsive expression, most *HAT* and *HDAC* promoters carry the auxin-response elements (AuxREs). In line with this finding, the *HAT/HDAC* genes were co-expressed with the *ARF*s (*AUXIN RESPONSE FACTOR*) which are core regulators in auxin signalling and make a fundamental contribution to embryogenic induction (Tables S9 and S10; PlantPan 3.0, [115]) [116].

The impaired embryogenic response of numerous *hat (hag2*, *hac1*, *hac4*, *hac5, haf1* and *haf2)* and *hdac* (*hda2*, *hda5*, *hda7*, *hdt1*, *hdt3*, *hdt4* and *srt1)* mutants suggests that the relevant HAT/HDACs positively control the SE response (Figure 4 and Figure 7). Within them, the function of the *HDT1, HDT2, HDT3, HDT4* and *HDA7* of *HDAC*s and *HAC1* of the *HAT*s in embryogenic development both in vivo and in vitro has been postulated [29,117,118,119]. The SE-related functions of other *HDAC*s (*HDA5, HDA9* and *SRT1*) and *HAT*s (*HAG4*, *HAG5*) might also be assumed, given the contribution of these genes to plant flowering and the ethylene responses of transcriptomic similarity to SE induction [63,120,121,122,123].

In contrast to the majority of *hat/hdac* mutants, the *gcn5–1*/*hag1–5* mutants and the *HDA19:RNAi* transgenic line had an improved embryogenic potential, thus suggesting that the relevant genes, *HAG1/GCN5* and *HDA19*, negatively control SE induction (Figure 5 and Figure 8). Similarly, the silencing of *HDA19* resulted in the formation of somatic embryo-like structures on the germinating seedlings [37]. In line with their assumed roles in SE induction, HAG1/GCN5 and HDA19 control various developmental processes and stress responses in plants, including cell differentiation and hormone responses [8,124,125].

We provide experimental evidence that *HDA19* might control the expression of *TF*s during SE, including negative *LEC1* and *LEC2* regulation and a positive impact on *BBM* (Figure 9A). Similarly, the HDA19-mediated negative control of *LEC2* has also been indicated in the seedlings of Arabidopsis [57]. In addition to *LEC2*, HDA19 has been postulated to control *LEC1* in seedling development, although ambiguous results have also been reported on HDA19 binding to *LEC1* [57,126]. The ChIP results indicated that the increase in the *LEC2* expression in the SE-induced explants was associated with an H3ac accumulation in the gene-bound chromatin fragment (Figure 9C).

Our Hac analyses involved a chromatin fragment that was bound to the TSS+300 bp gene region with an indicated role in the Hac-mediated control of genes [4,5]. A lack of Hac changes in the TSS+300 bp fragment of *LEC1* and *BBM* does not exclude other gene regions, including the promoter that is involved in HDA19-controlled Hac [57]. Another scenario for the HDA19-mediated gene regulation might involve the indirect effect of HDA19 on the *LEC1* and *BBM* expression, possibly via *LEC2*. In support of this hypothesis, the complex direct and indirect regulatory interactions between LEC2 and the other TFs, including LEC1 and BBM, in SE induction have been documented [116].

In order to control plant development, HAG1/GCN5 and HDA19 might affect the versatile gene regulatory pathways and many transcripts were deregulated in the *gcn5* and *hda19* mutants [127,128]. The results of the *TFs* profiling in the *gcn5-1* culture showed a deregulation of the *LEC1*, *LEC2* and *BBM* expression during SE (Figure S4B). Both a positive and negative regulation of gene expression via HAG1/GCN5 has been reported and its targets also involve the genes that are involved in SE [26,116,129]. Because many transcripts were deregulated in the *gcn5* and thousands of gene promotors might be HAG1/GCN5-targeted [130], the correct interpretation of the results regarding the deregulation of the genes in the *gcn5-1* culture (Figure S4B) in terms of the regulatory relationship between HAG1/GCN5 and *TF*s in SE, requires further analysis. In particular, the role of HAG1/GCN5 in the early stage of SE induction (before the fifth day) might be important for identifying how HAG1/GCN5 controls SE. In support of this postulate is the effect of a specific HAG1/GCN5 inhibitor, MB-3 [131], on the early embryogenic response of the explants (JM and MDG unpublished results). The results showed that the MB-3-induced inhibition of HAG1/GCN5 that occurred at the early stage of the culture severely impaired the SE response of the explants. Thus, a complex and SE stage-specific effect of HAG1/GCN5 on the target gene expression might be assumed, including a cooperative activity with other HAT/HDACs such as HDA19 [124,132]. Moreover, the Hac-mediated regulatory network that controls the *TF*s during SE might also involve HDT1 and HDT4 (Figure S4A). In support of this, the role of HDT1 in regulating *BBM* during callus formation and somatic cell reprogramming has also been postulated [25]. 

## 5. Conclusions

**Figure 10:**
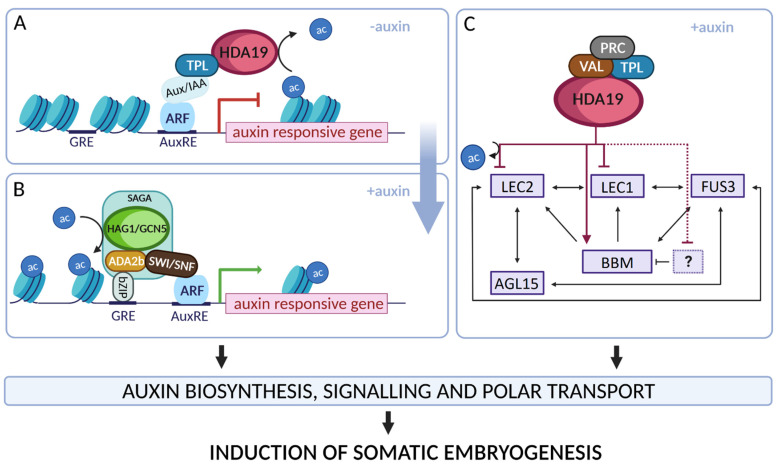
A model of the Hac-mediated control of auxin-responsive genes, including master regulators of SE induction. To regulate the auxin-responsive genes, histone acetyltransferases and histone deacetylases such as HDA19 (**A**) and HAG1/GCN5 (**B**) are assumed to cooperate with the other regulatory proteins of the transcriptional complexes. HDA19 contribute to SE induction by controlling the TFs encoding genes that have a master regulatory function during SE (**C**). ac—acetyl group; ADA2b—adapter protein; AGL15—AGAMOUS-LIKE 15; ARF—AUXIN RESPONSE FACTOR; Aux/IAA—Auxin/INDOLE-3-ACETIC ACID; AuxRE—Auxin RESPONSE ELEMENT; BBM—BABY BOOM; bZIP—bZIP-RELATED BASIC LEUCINE ZIPPER; FUS3—FUSCA3; GRE—G-BOX RELATED ELEMENT; HAG1/GCN5—HISTONE ACETYLTRANSFERASE OF THE GNAT FAMILY 1/GENERAL CONTROL NON-REPRESSED PROTEIN 5; HDA19—HISTONE DEACETYLASE 19; LEC1;2—LEAFY COTYLEDON 1; 2; PRC—POLYCOMB REPRESSIVE COMPLEX; SAGA—Spt/Ada/GCN5-ACETYLTRANSFERASE; SWI/SNF—SWITCH/SUCROSE NON-FERMENTING; TPL—TOPLESS; VAL—VIVIPAROUS1/ABI3-LIKE. Regulatory interactions that require further experimental validation are marked with a dashed line. The schemata was created using BioRender.com.

Consistent with the central role of auxin in SE induction, a model for the Hac-mediated control of the auxin-responsive genes, including the *TF*s that are essential in SE induction, is postulated (Figure 10). In support of the relevance of the model to SE-*TF*s, AuxRE and GRE elements were identified in the promoters of *LEC1, LEC2* and *BBM* (PlantPan 3.0, [115]). Following the model, in the absence of auxin, the HDA19-TPL repressive complex binds the AuxRE elements and downregulates the target auxin-responsive genes via interactions with Aux/IAA and the ARFs (Figure 10A) [133,134,135]. In turn, an accumulation of auxin results in the degradation of Aux/IAA, the release of the HDA19-TPL repressive complex and the recruitment of the SAGA co-activator complex with HAG1/GCN5 to the GRE motifs. As a result, the histones are acetylated and the transcription of the auxin-responsive gene is activated (Figure 10B) [93,136]. The auxin-dependent function of HDA19 in controlling the SE-involved *TF*s is postulated (Figure 10C).

Further progress in revealing the complex epigenome and transcriptome interactions during SE requires the regulatory proteins that recruit HAT/HDACs to the target loci to be identified, including HAG1/GCN5 and HDA19. The candidates involve AGL15, which has complex regulatory interactions with LECs and BBM during SE [39]. By cooperating with the TOPLESS co-repressors, AGL15 might enrol HDA6 and HDA19 into the transcriptional complexes in order to repress the target genes in plant development, including during SE [61,137].

## Data Availability

Not applicable.

## Supplementary Materials

The following are available online at https://www.mdpi.com/article/10.3390/cells11050863/s1, Table S1: The *hat* mutants used in the study, Table S2: The *hdac* mutants and RNAi transgenic lines used in the study, Table S3: Summary of the culture combinations and methods used in the study, Table S4: ChIP-qPCR analysis: sequence of the gene-specific primers, size of the amplicons and localisation of the analysed gene regions, Table S5: The gene-specific primers used for the RT-qPCR, Table S6: Comparison of the mean Alexa 488 and DAPI fluorescence intensity in the longitudinal sections across cotyledons of the Col-0 explants that had been cultured on the E0, EA and ET media for 0, 5 and 10 days, Table S7: The *HAT* gene expression patterns in the Col-0 explants that had been cultured on the E0, EA and ET media for 5 and 10 days, Table S8: The *HDAC* gene expression patterns in the Col-0 explants that had been cultured on the E0, EA and ET media for 5 and 10 days, Table S9: The promoter and co-expression analysis of the *HAT* genes, Table S10: The promoter and co-expression analysis of the *HDAC* genes, Figure S1: The immunodetected spatio-temporal pattern of H3ac in the cotyledons of the Col-0 explants, Figure S2: The H3ac and H4ac levels and expression profiles of *BBM*, *AGL15* and *WUS* in the Col-0 explants that had been cultured on the E0, EA and ET media for 5 and 10 days, Figure S3: The embryogenic capacity of the *hat* mutants on the ET medium evaluated in the 21-day-old culture, Figure S4: The expression profiles of the SE-*TF*s in the *hdt1*, *hdt4* and *gcn5-1* mutants in the freshly isolated (0 d) and explants that had been cultured on the EA medium (5 and 10 d).
