# Supplementary material for: Insights into the Histone Acetylation-Mediated Regulation of the Transcription Factor Genes That Control the Embryogenic Transition in the Somatic Cells of Arabidopsis
Source: Cells. 2022 Mar 2;11(5):863. doi: 10.3390/cells11050863 (PMC8909028; doi:10.3390/cells11050863)
Supplement: Supplementary file 1 [file cells-11-00863-s001.zip › cells-1529452-supplementary.pdf]

cells MDPI

Supplementary Materials

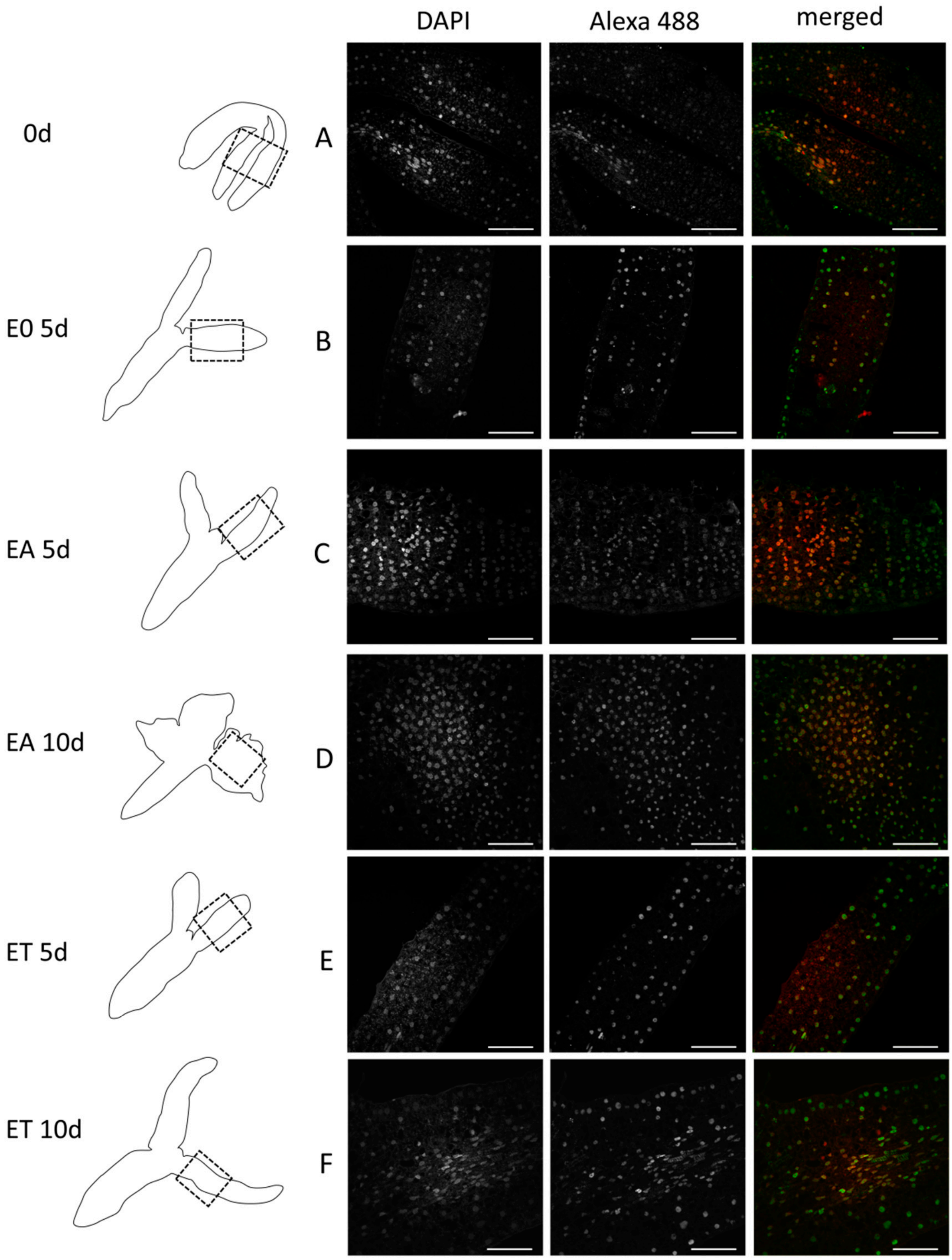

Figure S1. The immunodetected spatio-temporal pattern of H3ac in the cotyledons of the Col-0 explants. The explants were freshly isolated (0 d) (**A**) and *in vitro* cultured for 5 and 10 days on different media, including E0 (**B**), EA (**C**, **D**) and ET (**E**, **F**). Dashed line squares indicate explant areas in which Alexa 488 (H3ac) and DAPI (DNA) fluorescence intensity were measured. Red (computer altered)—DAPI; green (computer altered)—Alexa 488 (immunostaining of H3ac). Scale bar represents 50 μm.

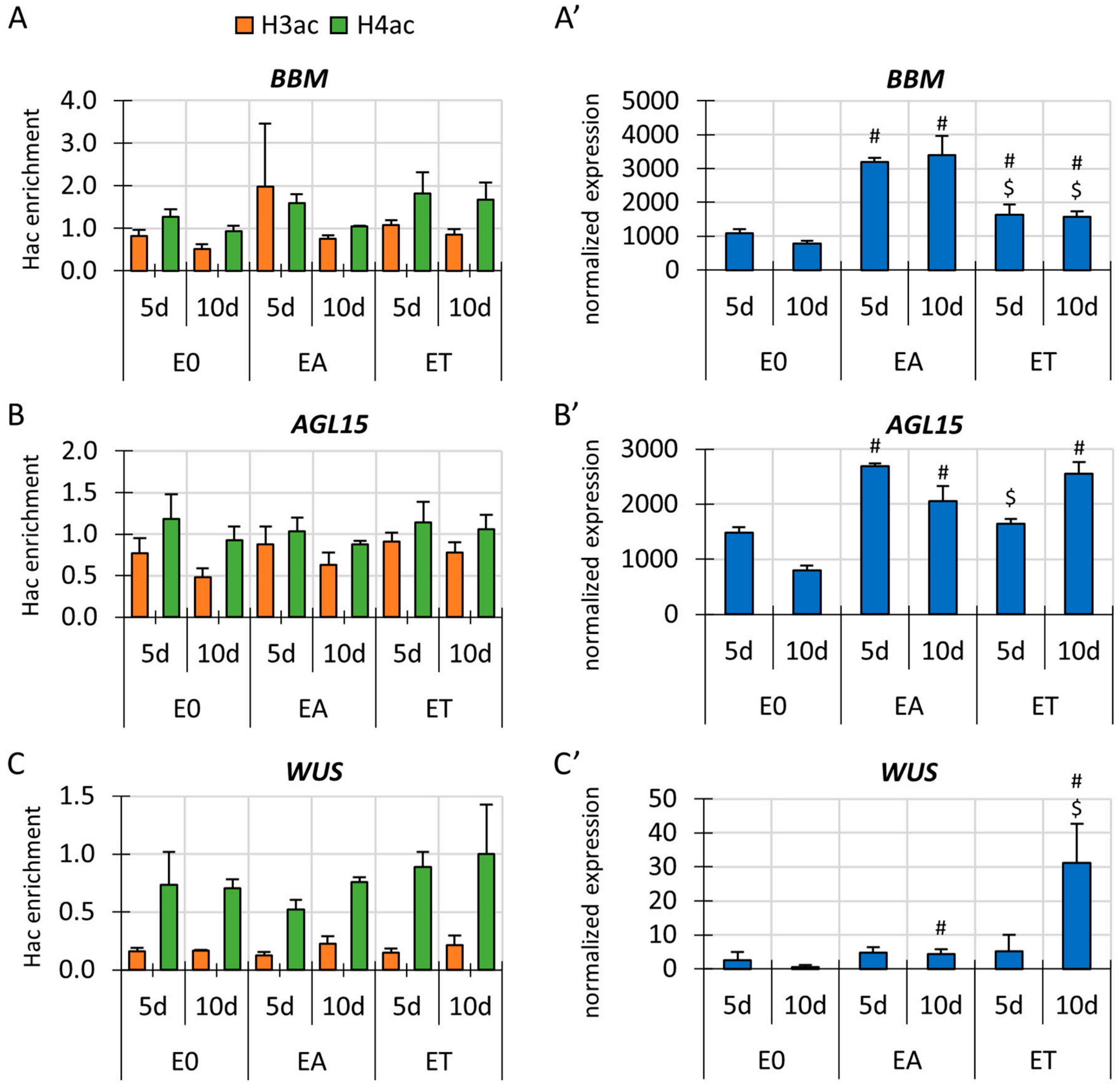

Figure S2. The H3ac and H4ac levels and expression profiles of *BBM* (**A**,**A'**), *AGL15* (**B**,**B'**) and *WUS* (**C**,**C'**) in the Col-0 explants that had been cultured on the E0, EA and ET media for 5 and 10 days. Hac enrichment indicates the amount of DNA after ChIP that was normalised to the internal control (*ACTIN7*). ChIP-qPCR: a two-way ANOVA analysis ($p < 0.05$) followed by Tukey's HSD test ($p < 0.05$) were used to determine the values that were significantly different from the E0 culture at the same age (#); the EA culture at the same age ($) (n = 3; means are given ± SD). RNA-seq: Wald's exact test was used to identify any differentially expressed genes (DEGs) under the *p*-value adjustment ($p < 0.05$) for multiple comparisons with the Benjamini-Hochberg False Discovery Rate (FDR) correction. Values that were significantly different from the E0 culture at the same age (#); the EA culture at the same age ($) (n = 3; means are given ± SD).

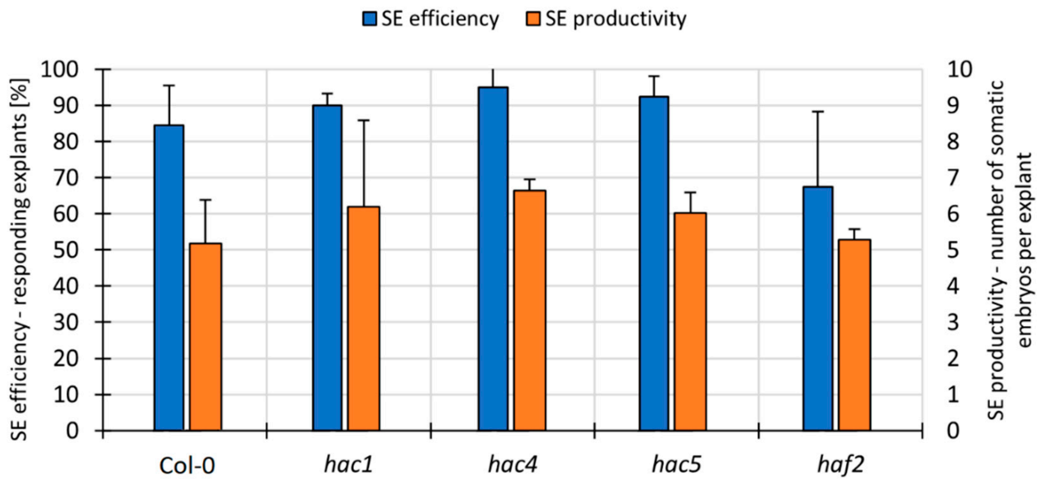

Figure S3. The embryogenic capacity of the *hat* mutants on the ET medium, evaluated in the 21-day-old culture. No significantly different values from the control WT culture (Col-0) were observed (n ≥ 3; means ± SD are given) (Student's t-test, $p < 0.05$).

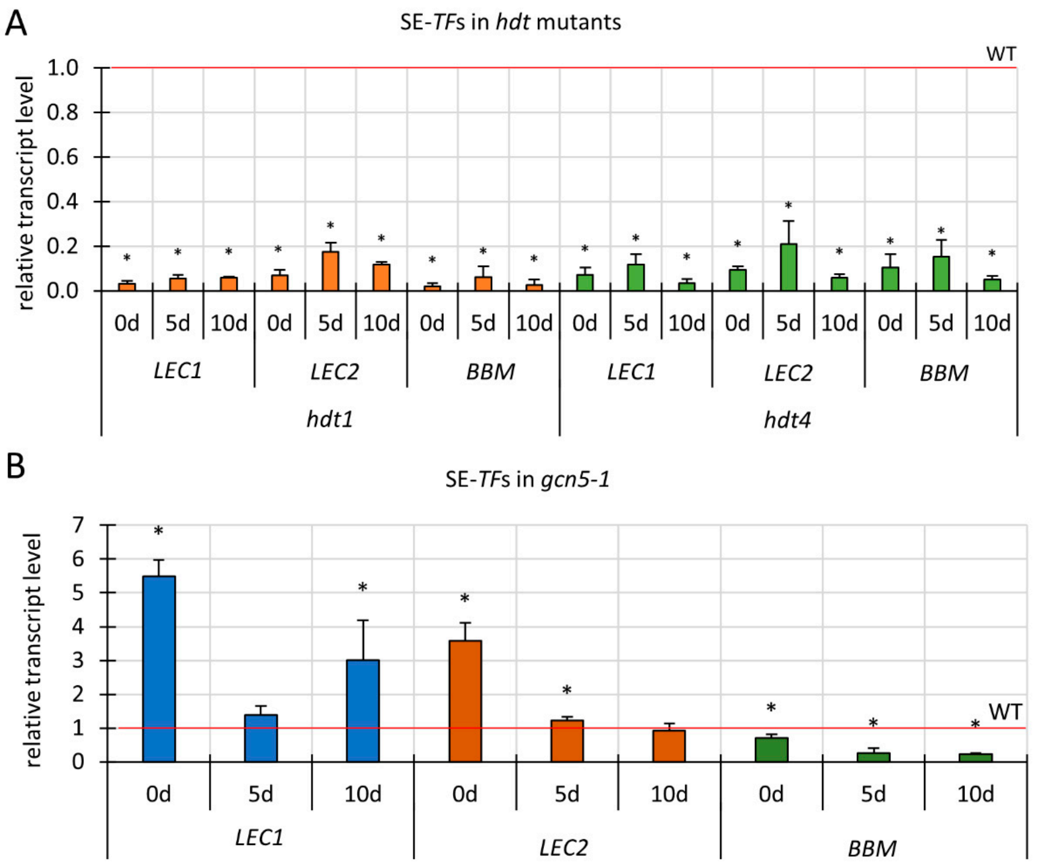

Figure S4. The expression profiles of the SE-*TFs* in the *hdt1*, *hdt4* (**A**) and *gcn5-1* (**B**) mutants in the freshly isolated (0 d) and explants that had been cultured on the EA medium (5 and 10 d). The relative transcript level was normalized to the internal control (*TIN* gene) and calibrated to the control WT cultures of Col-0 (A) and Ws-2 (B). Graph represents the data from the RT-qPCR analysis. An asterisk indicates an expression level that was significantly different from the one observed in WT (n = 3; means ± SD are given) (Student's t-test, $p < 0.05$).

Table S1. The *hat* mutants used in the study.

Gene	AGI	ID	Mutant	Insertion site	Source of seeds
HAG1/GCN5	AT3G54610	SALK_048427C	*hag1-5*	9th intron	NASC
		-	*gcn5-1* (Ws-2 background)	13th exon	[1]
HAG2	AT5G56740	SALK_051832C	*hag2-1*	promoter/exon	NASC
HAG3	AT5G50320	SALK_104121	*hag3*	3'UTR	NASC
HAG4	AT5G64610	SALK_027726C	*hag4*	1st exon	NASC
HAG5	AT5G09740	SALK_106046	*hag5*	2nd intron	NASC
HAC1	AT1G79000	SALK_122894	*hac1*	14st exon	NASC
HAC2	AT1G67220	SALK_049434C	*hac2-1*	promoter	NASC
HAC4	AT1G55970	SALK_006923	*hac4*	8th exon	NASC
HAC5	AT3G12980	SALK_152684	*hac5*	5'UTR/exon	NASC
HAC12	AT1G16710	SALK_052490	*hac12-1*	15th intron	NASC
HAF1	AT1G32750	SALK_110848C	*haf1-1*	20th exon	NASC
HAF2	AT3G19040	SALK_088103C	*haf2*	10th intron	NASC

[1] Seeds kindly provided by Prof. Konstantinos Vlachonasios, School of Biology, Aristotle University of Thessaloniki, Thessaloniki, Greece.

Table S2. The *hdac* mutants and RNAi transgenic lines used in the study.

Gene	AGI	ID	Mutant/transgenic line	Insertion site	Source of seeds
HDA2	AT5G26040	SALK_041074C	*hda2*	1st exon	[1]
HDA5	AT5G61060	SALK_007503	*hda5-1*	12th intron	[2]
HDA6	AT5G63110	CS24039	*HDA6:RNAi* (Ws-2 background)	-	NASC
HDA7	AT5G35600	SALK_002912C	*hda7*	promoter	[1]
HDA8	AT1G08460	GK_100A06	*hda8*	1st exon	[1]
HDA9	AT3G44680	SALK_007123	*hda9-1*	5th exon	[2]
HDA14	AT4G33470	SALK_097005C	*hda14*	4th intron	NASC
HDA15	AT3G18520	SALK_004027	*hda15-1*	2nd exon	[2]
HDA18	AT5G61070	SALK_006938	*hda18-1*	7th exon	NASC
HDA19	AT4G38130	CS30925	*HDA19:RNAi* (Ws-2 background)	-	NASC
HDT1	AT3G44750	GK_355H03	*hdt1*	3rd exon	[1]
HDT2	AT5G22650	SAIL_1247_A02	*hdt2*	promoter	[1]
HDT3	AT5G03740	SALK_129799C	*hdt3-1*	5th intron	NASC
HDT4	AT2G27840	GABI_836B08	*hdt4*	5'UTR	[1]
SRT1	AT5G55760	SALK_001493	*srt1*	14th exon	NASC
SRT2	AT5G09230	SALK_149295	*srt2*	2nd exon	NASC

Seeds kindly provided by:.
[1] Dr. Kim Boutilier, Bioscience, Wageningen University and Research, Wageningen, Netherlands;.
[2] Prof. Keqiang Wu, Institute of Plant Biology, National Taiwan University, Taipei, Taiwan.

Table S3. Summary of the culture combinations and methods used in the study.

Method		Analysed samples								
		0d	E0			EA			ET	
			5d	10d	21d	5d	10d	21d	5d	10d
	ELISA total H3ac	x	x	x		x	x		x	x
	Immunodetection of H3ac	x	x			x	x		x	x
	RNA-seq	x	x	x		x	x		x	x
SE response	*hag1-5, gcn5-1* and *HDA19:RNAi*				x			x		
	other *hat/hdac* mutants							x		
	ChIP-qPCR		x	x		x	x		x	x
	RT-qPCR	x				x	x			
	ChIP-qPCR in *HDA19:RNAi* line					x				

Table S4. ChIP-qPCR analysis: sequence of the gene-specific primers, size of the amplicons and localisation of the analysed gene regions.

Gene	AGI	pF [5' - 3']	pR [5' - 3']	Amplicon size [bp]	Analyzed region
LEC1	AT1G21970	TGAGACTTTGCAACAACGAACT	AAAATCGTCTTCTCCGGGCC	95	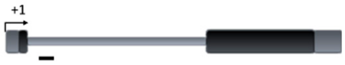
		CGGCAGAGAAACAATGGAAC [1]	CAGAGATGGATTTTGGTAGCTG [1]	65	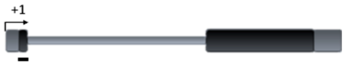
LEC2	AT1G28300	CCGTTTTCATACCCGGTGGA	TTGCGTAACCGGGATCTGAG	93	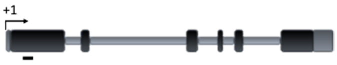
FUS3	AT3G26790	TTCGATCTGCACAACACACA	TGGTGGATCTCTTTGGGTTTCA	119	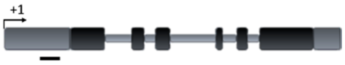
AGL15	AT5G13790	TTTGGGGGAAAATGGGTCGT	CCCAGAACGCCTCTTGGAAA	92	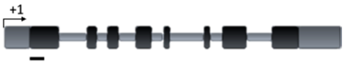
MYB118	AT3G27785	AGGGTTTCATGCTTCACTCA	TGAACACTGACTCGAACTCCA	110	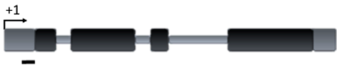
BBM	AT5G17430	AGGCTTCTCTCTCTCTCCTCA	AACATCTACGGCGGTTCTGG	82	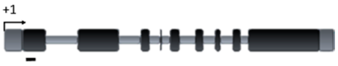
WUS	AT2G17950	GCCACAGCATCAGCATCATC	CGACACGTGTAACCACCAGA	90	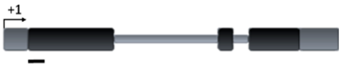
ACTIN7 [2]	AT5G09810	CGTTTCGCTTTCCTTAGTGTTAGCT	AGCGAACGGATCTAGAGACTCACCTTG	134	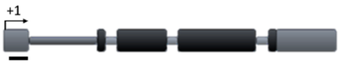

[1] – used for ChIP-qPCR in Ws-2 and *HDA19:RNAi* line.
[2] – according to Luo et al. [63].

Table S5. The gene-specific primers used for the RT-qPCR.

Gene	AGI	pF [5′ - 3′]	pR [5' - 3']
LEC1	AT1G21970	GTGGAGCTCCCTTCTCTCACT	CTGGACCACGATACCATTGTT
LEC2	AT1G28300	AGGGAAAGGAACCACTACGAA	CAGTGGTGAGGTCCATGAGAT
BBM	AT5G17430	AATGCTAATCATCAAGACAAT	ATCTACCTGTCCACCGATGC
TIN	AT4G27090	GTCGTTATCGTCGACGTTGTT	CCTCGATCAAAGCCTTCTTCT

Table S6. Comparison of the mean Alexa 488 and DAPI fluorescence intensity in the longitudinal sections across cotyledons of the Col-0 explants that had been cultured on the E0, EA and ET media for 0, 5 and 10 days.

		Replicate	Alexa 488 fluorescence		DAPI fluorescence		Alexa 488/DAPI fluorescence
			Mean	No. of nuclei	Mean	No. of nuclei	
0d		1	336	154	1301	254	0.26
		2	533	132	851	404	0.63
		3	246	27	579	359	0.42
E0	5d	1	804	85	1926	154	0.42
		2	598	266	1495	207	0.40
		3	719	137	1319	85	0.54
		4	666	322	881	261	0.76
EA	5d	1	467	378	1344	402	0.35
		2	707	441	1238	651	0.57
		3	948	190	1382	316	0.69
		4	463	629	796	642	0.58
	10d	1	472	923	877	742	0.54
		2	634	1983	666	1917	0.95
ET	5d	1	973	554	1977	464	0.49
		2	1209	322	1913	278	0.63
		3	533	1029	996	811	0.54
	10d	1	1215	255	1694	100	0.72
		2	1039	606	1469	443	0.71
		3	710	1930	948	2055	0.75

Table S7. The *HAT* gene expression patterns in the Col-0 explants that had been cultured on the E0, EA and ET media for 5 and 10 days.

Gene family	Gene name	Gene ID	A				B			
			EA vs. E0				ET vs. E0			
			5d		10d		5d		10d	
			FC	*p*-value	FC	*p*-value	FC	*p*-value	FC	*p*-value
GNAT	*HAG1/GCN5*	AT3G54610	1.2	1E-01	1.3	✓ 3E-02	1.0	1E+00	1.2	2E-01
	HAG2	AT5G56740	1.8	✓ 5E-05	1.5	✓ 5E-03	2.2	✓ 4E-08	1.8	✓ 6E-05
	HAG3	AT5G50320	1.1	1E-01	1.3	✓ 3E-03	1.1	6E-01	1.1	2E-01
MYST	*HAG4*	AT5G64610	1.6	✓ 4E-03	1.2	5E-01	1.2	5E-01	0.9	5E-01
	HAG5	AT5G09740	1.3	1E-01	1.4	✓ 4E-02	1.0	1E+00	1.2	2E-01
CBP	*HAC1*	AT1G79000	1.0	9E-01	1.0	1E+00	0.8	3E-01	0.8	2E-01
	HAC2	AT1G67220	1.8	3E-01	5.9	✓ 9E-04	2.4	1E-01	12.6	✓ 1E-06
	HAC4	AT1G55970	1.2	4E-01	1.0	1E+00	0.7	1E-01	1.0	9E-01
	HAC5	AT3G12980	1.3	✓ 1E-02	1.1	4E-01	0.9	4E-01	0.9	2E-01
	HAC12	AT1G16710	1.1	4E-01	1.1	4E-01	0.9	6E-01	0.9	4E-01
TAF$_{II}$250	*HAF1*	AT1G32750	1.0	9E-01	1.3	3E-01	1.3	5E-01	1.2	5E-01
	HAF2	AT3G19040	1.0	1E+00	1.1	8E-01	3.8	✓ 1E-03	2.0	1E-01

Values represent the relative expression level (fold change, FC) in EA vs. E0 (**A**) and ET vs. E0 (**B**) media at the 5th and 10th day of the culture. Data from the RNA-seq analysis are given. Wald's exact test was used to identify any differentially expressed genes (DEGs) under a *p*-value adjustment ($p < 0.05$) for multiple comparisons with the Benjamini-Hochberg False Discovery Rate (FDR) correction. Significantly different values are indicated with a checkmark.

Table S8. The HDAC gene expression patterns in the Col-0 explants that had been cultured on the E0, EA and ET media for 5 and 10 days.

Gene family	Gene name	Gene ID	A				B			
			EA vs. E0				ET vs. E0			
			5d		10d		5d		10d	
			FC	*p*-value	FC	*p*-value	FC	*p*-value	FC	*p*-value
RPD3/ HDA1	*HDA2*	AT5G26040	1.3	2E-01	0.7	9E-02	1.1	1E+00	0.7	1E-01
	HDA5	AT5G61060	1.6	✓ 8E-05	1.5	✓ 2E-03	1.8	✓ 6E-06	1.3	✓ 5E-02
	HDA6	AT5G63110	1.2	1E-01	1.1	6E-01	1.7	✓ 7E-09	1.3	✓ 1E-03
	HDA7	AT5G35600	1.4	NA	1.0	NA	0.6	8E-01	1.7	NA
	HDA8	AT1G08460	0.9	7E-01	1.1	8E-01	0.7	✓ 2E-02	1.1	5E-01
	HDA9	AT3G44680	1.4	✓ 3E-04	1.4	✓ 3E-04	1.4	✓ 5E-03	1.1	7E-01
	HDA10	AT3G44660	1.0	NA	1.0	NA	1.0	1E+00	0.9	NA
	HDA14	AT4G33470	1.3	3E-01	0.9	4E-01	1.0	1E+00	0.7	7E-02
	HDA15	AT3G18520	1.3	✓ 4E-02	1.4	✓ 7E-03	1.3	6E-02	1.2	2E-01
	HDA17	AT3G44490	1.5	NA	0.9	NA	0.8	1E+00	1.0	1E+00
	HDA18	AT5G61070	1.4	5E-01	1.7	2E-01	4.7	✓ 2E-04	7.5	✓ 2E-07
	HDA19	AT4G38130	1.2	3E-01	1.6	✓ 7E-03	1.4	9E-02	1.5	✓ 2E-02
HD2	*HDT1*	AT3G44750	1.9	✓ 2E-10	1.6	✓ 4E-06	2.1	✓ 6E-12	1.0	8E-01
	HDT2	AT5G22650	1.5	✓ 5E-02	1.5	✓ 2E-02	2.5	✓ 7E-07	1.3	2E-01
	HDT3	AT5G03740	1.6	✓ 1E-03	1.3	7E-02	2.2	✓ 7E-09	2.0	✓ 1E-07
	HDT4	AT2G27840	1.4	1E-01	1.1	8E-01	1.8	✓ 1E-02	0.7	1E-01
SIR2	*SRT1*	AT5G55760	1.5	✓ 4E-03	1.4	✓ 2E-02	1.6	✓ 8E-04	1.5	✓ 1E-03
	SRT2	AT5G09230	1.1	6E-01	1.0	1E+00	0.9	5E-01	1.1	6E-01

Values represent the relative expression level (fold change, FC) in the EA vs. E0 (**A**) and ET vs. E0 (**B**) media at the 5th and 10th day of culture. Data from the RNA-seq analysis are given. Wald's exact test was used to identify any differentially expressed genes (DEGs) under a *p*-value adjustment ($p < 0.05$) for multiple comparisons with the Benjamini-Hochberg False Discovery Rate (FDR) correction. Significantly different values are indicated with a checkmark.

Table S9. The promoter and co-expression analysis of the *HAT* genes.

HAT	AuxRE [1]	TFBS for SE-*TFs* [2,3]	Co-expression with *ARF*s		Co-expression with SE-*TFs* [3]	
			Environmental stress	Hormone treatment	Environmental stress	Hormone treatment
HAG1/ GCN5	YES	*LEC1, FUS3, MYB118, WUS*	-	*ARF4, ARF6, ARF8*	-	-
HAG2	NO	*LEC1, AGL15*	-	-	*LEC1, AGL15*	*AGL15*
HAG3	YES	*LEC1, AGL15, WUS*	*ARF4*	*ARF4, ARF8*	*AGL15*	*AGL15*
HAG4	YES	*LEC1, AGL15*	*ARF1*	*ARF1, ARF4, ARF8, ARF11, ARF16, ARF17, ARF18*	-	*AGL15*
HAG5	YES	*LEC1, MYB118, WUS*	*ARF1, ARF4, ARF6, ARF8, ARF13, ARF17, ARF21*	-	*LEC1, WUS*	-

HAC1	YES	*LEC1, FUS3*	-	*ARF12*	-	-
HAC2	YES	*LEC1, AGL15, WUS*	*ARF4, ARF6, ARF8, ARF11*	-	*LEC1, AGL15, WUS*	-
HAC4	YES	*LEC1, AGL15*	-	*ARF16*	*LEC1, AGL15*	*AGL15*
HAC5	YES	*LEC1, LEC2, FUS3, AGL15, MYB118, WUS*	-	*ARF4, ARF8*	*AGL15*	*AGL15*
HAC12	YES	*LEC1, MYB118*	*ARF1, ARF2, ARF4, ARF6, ARF8, ARF13, ARF17, ARF21, ARF23*	*ARF1, ARF4, ARF8, ARF11, ARF16, ARF17, ARF18*	*LEC1*	-
HAF1	YES	*LEC1, AGL15, WUS*	-	*ARF4, ARF8, ARF16*	-	-
HAF2	YES	*LEC1, MYB118, WUS*	-	-	*LEC1, WUS*	-

[1] – AuxRE – auxin-response elements.
[2] – TFBS – transcription factor binding site.
[3] – SE-*TF*s analysed in the study.

Table S10. The promoter and co-expression analysis of the *HDAC* genes.

HDAC	**AuxRE** [1]	**TFBS for SE-*TFs*** [2, 3]	**Co-expression with *ARF*s**		**Co-expression with SE-*TFs*** [3]	
			**Environmental stress**	**Hormone treatment**	**Environmental stress**	**Hormone treatment**
HDA2	YES	*LEC1*	*ARF1, ARF4, ARF8, ARF10, ARF17*	*ARF1, ARF4, ARF8, ARF10, ARF16, ARF18*	-	-
HDA5	YES	*LEC1, LEC2, FUS3, AGL15*	*ARF4, ARF6, ARF8, ARF13, ARF17, ARF21, ARF23*	*ARF1, ARF8, ARF16, ARF18, ARF48*	*LEC1, LEC2, AGL15*	-
HDA6	YES	*LEC1, WUS*	*ARF4, ARF6, ARF8, ARF11*	-	*LEC1, WUS*	-
HDA7	YES	*LEC1, LEC2, FUS3, WUS*	-	-	*LEC1, LEC2, WUS*	-
HDA8	YES	*LEC1, AGL15*	-	-	*LEC1, AGL15*	-
HDA9	YES	*LEC1*	*ARF1, ARF4, ARF17*	*ARF1, ARF4, ARF6, ARF8,*	-	-

				ARF11, ARF16, ARF17, ARF18		
HDA10	NO	*LEC1, MYB118, WUS*	-	-	-	-
HDA14	NO	*LEC1, LEC2, MYB118, WUS*	-	-	-	-
HDA15	YES	*LEC1, LEC2, FUS3, MYB118, WUS*	*ARF4, ARF6, ARF8, ARF11*	-	*LEC1, LEC2, FUS3, WUS*	-
HDA17	NO	*LEC1, MYB118, WUS*	-	-	-	-
HDA18	YES	*LEC1, WUS*	*ARF2, ARF4, ARF6, ARF8, ARF12, ARF13, ARF21, ARF23*	-	*LEC1, WUS*	-
HDA19	YES	*LEC1, MYB118, WUS*	*ARF4, ARF6, ARF8, ARF11, ARF12, ARF13, ARF17, ARF21, ARF23*	*ARF6, ARF11, ARF17*	*LEC1, WUS*	-
HDT1	YES	*LEC1*	*ARF1*	*ARF1, ARF4, ARF6, ARF8, ARF11, ARF16, ARF17, ARF18*	-	-
HDT2	YES	*LEC1, AGL15, MYB118*	*ARF1, ARF4, ARF6, ARF8, ARF10, ARF17*	*ARF1, ARF4, ARF6, ARF8, ARF11, ARF16, ARF17, ARF18*	*AGL15*	*AGL15*
HDT3	NO	*LEC1, MYB118*	-	-	-	-
HDT4	YES	*LEC1, AGL15*	-	-	-	-
SRT1	NO	*LEC1, FUS3*	-	-	*LEC1, LEC2, FUS3*	-
SRT2	NO	*LEC1, WUS*	-	-	*LEC1, WUS*	-

[1] – AuxRE – auxin-response elements.
[2] – TFBS – transcription factor binding site.
[3] – SE-*TF*s analysed in the study.
